# Geographically weighted Weibull regression modeling on dissolved oxygen data to analyze river water quality in East Kalimantan

**DOI:** 10.1016/j.mex.2025.103745

**Published:** 2025-11-30

**Authors:** Suyitno Suyitno, Darnah, Memi Nor Hayati, Andrea Tri Rian Dani, Ika Purnamasari, Rito Goejantoro, Meiliyani Siringoringo, Pratama Yuly Nugraha, Meirinda Fauziyah, Zabrina Nathania Fauziyah, Mislan

**Affiliations:** a Statistics Study Program, Department of Mathematics, Faculty of Mathematics and Natural Sciences, Mulawarman University; b Physics Study Program, Department of Physics, Faculty of Mathematics and Natural Sciences, Mulawarman University

**Keywords:** Bandwidth, GWWR model, River water quality, Spatial heterogeneity data, Weibull regression model extension

## Abstract

This study introduces the Geographically Weighted Weibull Regression (GWWR) model as an extension of the Weibull regression (WR) within the geographically weighted regression framework and applies it to spatial environmental data on dissolved oxygen (DO) levels in East Kalimantan in 2024, rather than to time-to-event data. This study maps the river water quality (RWQ) and its influencing factors using the GWWR model. The results indicate that the RWQ in East Kalimantan in 2024 generally tends to degrade, with the main influencing factors being dissolved iron, total phosphate, water temperature, and biochemical oxygen demand. The main highlights of the proposed method are as follows:

• This study presents the GWWR model as an extension of the WR model and demonstrates its applicability to spatially heterogeneous data rather than to time-to-event data.
• The GWWR model is employed to locally analyze RWQ and its influencing factors.
• The GWWR approach represents RWQ characteristics using several statistical measures, including the probability of water quality improvement, the probability of water quality degradation, the water quality degradation rate, and the mean DO level. These statistical measures are analyzed respectively through spatial Weibull survival, cumulative distribution, hazard, and mean regression models.

## Graphical abstract

**Figure:**
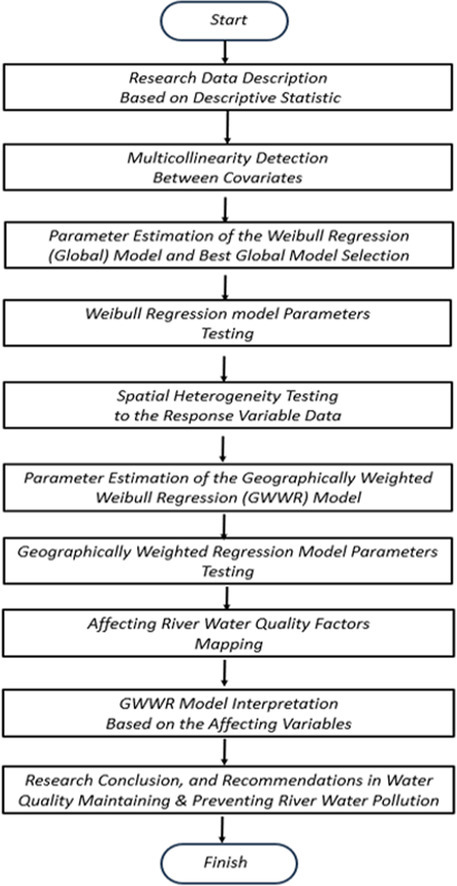

Related research article

1. Suyitno, Darnah, A.T.R. Dani, and N.T. Oktavia, “ Modeling the hospitalization time of stroke patients at Abdul Wahab Sjahranie Hospital Samarinda using the Weibull Regression Model,” *MethodsX*, Vol. *14*, no. 103082, Juny 2025, 10.1016/j.mex.2024.103082.
2. Suyitno, and N.W.W. Sari, “Parameter estimation of mixed geographically weighted weibull regression model,” *Journal of Physics: Conference Series*, Institute of Physics Publishing, Aug. 2019. 10.1088/1742-6596/1277/1/012046.

For a published article:

1. Suyitno, Darnah, A.T.R. Dani, and N.T. Oktavia, “ Modeling the hospitalization time of stroke patients at Abdul Wahab Sjahranie Hospital Samarinda using the Weibull Regression Model,” *MethodsX*, Vol. 14, no. 103082, Juny 2025, 10.1016/j.mex.2024.103082.
2. Suyitno, and N.W.W. Sari, “Parameter estimation of mixed geographically weighted Weibull regression model,” *Journal of Physics: Conference Series*, Institute of Physics Publishing, Aug. 2019. 10.1088/1742-6596/1277/1/012046.

Specifications table

This table provides general information on your method.

**Table:** 

Subject area	Mathematics and Statistics
More specific subject area	*Survival Time Data Analysis; Weibull Regression Models; Geographically Weighted Regression Models; Environmental Health*
Name of your method	*Geographically Weighted Weibull Regression Model and Its Applications*
Name and reference of original method	1. Suyitno, Darnah, Dani, A.T.R. and Oktavia, N.T., “Modeling the hospitalization time of stroke patients at Abdul Wahab Sjahranie Hospital Samarinda using the Weibull Regression Model,” *MethodsX*, Vol. 14, no. 103082, Juny 2025, 10.1016/j.mex.2024.103082. 2. Suyitno and N. W. W. Sari, “Parameter estimation of mixed geographically weighted weibull regression model”, *Journal of Physics: Conference Series*, Institute of Physics Publishing, Aug. 2019, 10.1088/1742-6596/1277/1/012046.
Resource availability	The research data used in this study are secondary data obtained from the 2024 Surface Water Quality Monitoring Analysis Report published by the Environmental Agency of East Kalimantan Province. The dataset consists of 27 sampling points. The variables include the response variable, namely the dissolved oxygen (DO) levels of river water in East Kalimantan (Y), and the covariates, namely dissolved iron (Fe) $\left(X_{1}\right)$, total phosphate $\left(X_{2}\right)$, water temperature $\left(X_{3}\right)$, biochemical oxygen demand (BOD) $\left(X_{4}\right)$, nitrate $\left(X_{5}\right)$, ammonia $\left(X_{6}\right)$, oil and grease $\left(X_{7}\right)$, pH or potential of hydrogen $\left(X_{8}\right)$, total suspended solids (TSS) $\left(X_{9}\right)$, and water color degree $\left(X_{10}\right)$. The data analysis technique employed in this study is the geographically weighted Weibull regression model, and all computations were performed using GNU Octave (open-source software).

## Background

The geographically Weighted Weibull Regression (GWWR) model extends the conventional Weibull Regression (WR) model to account for spatial heterogeneity in the data [[1], [2], [3], [4]]. The GWWR model is a local variant of the WR model based on the geographically weighted regression (GWR) framework. Local models within the GWR framework outperform the conventional global models [5,6]. The WR model is widely used for modeling time-to-event data, which are frequently encountered in medical and health-related studies [[7], [8], [9], [10]]*.* However, many types of nonnegative continuous data in the environmental field also follow a Weibull distribution [[11], [12], [13]], and such data are often spatially heterogeneous and not time-to-event data in nature [1,[14], [15], [16]]. Therefore, this study proposes extending the WR model within the GWWR framework and applying it to spatially heterogeneous, nonnegative continuous data, such as dissolved oxygen (DO) levels in river water [17,18]. DO is a key parameter for assessing river water quality [19,20].

The purpose of this study is to map river water quality (RWQ) in East Kalimantan in 2024 and to identify its influencing factors using GWWR modeling based on DO data [17,19,21]. The drinking water supply for most residents of East Kalimantan is provided by the Regional Drinking Water Company (PDAM), with approximately 74.35% of the total raw water volume derived from river water, followed by 15.30% from reservoirs and 9.46% from groundwater. Various activities along the watershed have the potential to discharge waste into river flows, which can significantly degrade water quality [[22], [23], [24], [25]]. Therefore, continuous efforts are required to maintain RWQ and prevent pollution. In this study, the GWWR model is proposed as a statistical approach to map RWQ and its influencing factors. Providing information on RWQ and its influencing factors is expected to raise public awareness and encourage community participation in maintaining water quality [26]. This aligns with the mission of the East Kalimantan Provincial Environmental Agency, which seeks to promote active community participation in environmental protection and to minimize pollution and negative environmental impacts.

Using the GWWR model, RWQ characteristics are represented by statistical measures comprising the probability of river water quality improvement, the probability of water quality degradation, the water quality degradation rate, and the mean DO level. These statistical measures are analyzed using spatial Weibull survival, cumulative distribution, hazard, and mean regression models, respectively. Previous studies have analyzed water quality using various statistical approaches [17,18,23,25,27].

This study proposes a parameter estimation approach based on the maximum likelihood estimation (MLE) method by combining the parameter estimation procedures of the GWR model [28,29] and the WR model for right-censored time-to-event data [14,15]. The spatial weights are calculated using an adaptive Gaussian function, while the optimal bandwidth is determined using the Bayesian Information Criterion (BIC). In addition to using BIC for bandwidth selection, the model’s goodness-of-fit is further evaluated using generalized cross-validation (GCV), McFadden’s R², and mean absolute percentage error (MAPE) [[30], [31], [32]].

## Method details

In this section, the following topics are presented sequentially: the new formulation of the Weibull distribution and Weibull regression models, which differs from those in [14], parameter estimation for the Weibull regression (WR) model; testing of the WR model parameters; development of the WR model into the Geographically Weighted Weibull Regression (GWWR) model; parameter estimation for the GWWR model; and parameter testing for the GWWR model.

### Weibull regression model

To begin the discussion of the Weibull regression model, this section first introduces the scale–shape parametrization of the Weibull distribution and its associated functions [33,34]. The probability density function (PDF) of a nonnegative continuous random variable Y that follows the scale–shape version of the Weibull distribution is defined as

(1) $$f\left(y\right)=\frac{d}{dy}F\left(y\right)=\gamma \tau^{-\gamma }y^{\gamma -1}\text{exp}\left[-{\left(\frac{y}{\tau }\right)}^{\gamma }\right],y\geq 0;0<\gamma ,\tau <\infty ,$$

where F(y) is the cumulative distribution function (CDF), τ is the scale parameter, and γ is the shape parameter. By applying the transformation $\lambda =\tau^{-\gamma }$, the PDF in Eq. (1) can be written as

(2) $$f\left(y\right)=\lambda \gamma y^{\gamma -1}\text{exp}\left[-\lambda y^{\gamma }\right],$$

where λ is a new scale parameter, with y≥0 and 0<γ,λ<∞. The associated functions, including the CDF, survival function, and hazard function, can be derived from the PDF in Eq. (2). Referring to Eqs. (1) and (2), the CDF of the Weibull distribution can be obtained from its definition as

(3) $$F\left(y\right)=P\left(Y\leq y\right)=\int_{0}^{y}f\left(t\right)dt=1-\text{exp}\left[-\lambda y^{\gamma }\right].$$

The Weibull survival function can be obtained as the complement of the probability in Eq. (3), given by:

(4) $$S\left(y\right)=P\left(Y>y\right)=1-F\left(y\right)=\text{exp}\left[-\lambda y^{\gamma }\right].$$

Using Eqs. (2) and (4), the hazard function of the scale–shape parametrization of the Weibull distribution is defined as

(5) $$h\left(y\right)=\lim_{\Delta y\to 0}\frac{P(y<Y\langle y+\Delta y|Y\geq y)}{\Delta y}=\frac{f\left(y\right)}{S\left(y\right)}=\lambda \gamma y^{\gamma -1}.$$

The expected value (mean) of the random variable Y with PDF in Eq. (2) is given by

(6) $$\mu_{Y}=E\left(Y\right)=\int_{0}^{\infty }yf\left(y\right)dy=\lambda^{-1/\gamma }\Gamma \left(1+\frac{1}{\gamma }\right),$$

where Γ denotes the Gamma function [35]. The parameters of the Weibull distribution are commonly estimated using the maximum likelihood estimation (MLE) method, and the maximum likelihood (ML) estimators can be obtained numerically using the Newton–Raphson iterative procedure [7,36]. The Kolmogorov–Smirnov nonparametric test can be used to assess the goodness-of-fit of the observed data to the Weibull distribution [14,16].

Weibull regression is a regression model derived from the Weibull distribution, in which the scale parameter of the Weibull distribution is directly influenced by covariates [14,37]. Mathematically, the Weibull regression (WR) model can be formulated based on the associated functions in Eqs. (2) – (5), where the scale parameter λ is expressed as a function of the regression parameters and covariates. Specifically, since λ in Eq. (2) is positive, it is modeled as an exponential function of the regression parameters:

(7) $$\lambda \left(x\right)=\text{exp}\left(b^{T}x\right)=\text{exp}\left(b_{0}+b_{1}X_{1}+b_{2}X_{2}+\cdots +b_{p}X_{p}\right),$$

where $b^{T}=\left[\begin{array}{ccc} b_{0} & b_{1} & \begin{array}{cc} \cdots & b_{p} \end{array} \end{array}\right]$ and $x={\left[\begin{array}{ccc} X_{0} & X_{1} & \begin{array}{cc} \cdots & X_{p} \end{array} \end{array}\right]}^{T},\;X_{0}=1$. By substituting Eq. (7) into the survival function in Eq. (4), a new Weibull survival regression model is obtained, which can be expressed as

(8) $$S\left(y,x,\varphi \right)=S\left(y,x\right)=P\left(Y>y|x\right)=\text{exp}\left[-y^{\gamma }\text{exp}\left(b^{T}x\right)\right],$$

*where* $\varphi ={\left[\begin{array}{cc} \gamma & b^{T} \end{array}\right]}^{T}={\left[\begin{array}{ccc} \gamma & b_{0} & \begin{array}{ccc} b_{1} & \cdots & b_{p} \end{array} \end{array}\right]}^{T}$*, and* $b^{T}=\left[\begin{array}{ccc} b_{0} & b_{1} & \begin{array}{cc} \cdots & b_{p} \end{array} \end{array}\right]$*. The Weibull cumulative distribution regression model is obtained based on the relationships in* Eqs. (3)*, (*4*) and (*7*), which can be expressed as*

(9) $$F\left(y,x\right)=P\left(Y\leq y|x\right)=1-S\left(y,x\right)=1-\text{exp}\left[-y^{\gamma }\text{exp}\left(b^{T}x\right)\right].$$

Referring to Eqs. (5) and (7), the Weibull hazard regression model can be expressed as

(10) $$h\left(y,x\right)=\gamma y^{\gamma -1}\text{exp}\left(b^{T}x\right).$$

Similarly, the Weibull regression for the mean can be obtained based on Eqs. (6) and Eq. (7), which can be expressed as

(11) $$\mu_{Y}\left(x\right)=\Gamma \left(1+\frac{1}{\gamma }\right)\text{exp}\left(-\frac{1}{\gamma }b^{T}x\right).$$

By substituting Eq. (7) into Eq. (2), the PDF as influenced by the covariates is obtained, which can be expressed as

(12) $$f\left(y,x\right)=\left[\gamma y^{\gamma -1}\text{exp}\left(b^{T}x\right)\right]\text{exp}\left[-y^{\gamma }\text{exp}\left(b^{T}x\right)\right].$$

### Parameter estimation of the Weibull regression model

The parameter estimation method of the WR model in this study was MLE [38,39]. MLE is a commonly used parameter estimation method for non-linear regression models, such as Poisson regression, geographically weighted logistic regression, and multivariate Weibull regression [4,34,40]. While the WR is typically applied to right-censored time-to-event data, in this study it is applied to non-negative continuous data, not time-to-event data [5,6,41]. The response variable consists of observations that experience the event (complete observations) and right-censored observations at a censoring point $y^{*}$ for those that survive, with values defined as:

(13) $$y_{i}=\left\{ \begin{array}{c} y_{i\;},\;if\;y_{i}<y^{*}\\ y^{*},\;if\;y_{i}\geq y^{*} \end{array}\right..$$

The censoring status for the i-th observation $\left(\delta_{i}\right)$ is defined as:

(14) $$\delta_{i}=\left\{ \begin{array}{c} 1,\;if\;y_{i}<y^{*}\\ 0,\;if\;y_{i}\geq y^{*} \end{array}\right..$$

Suppose an independent sample $(y_{i},x_{i},\delta_{i}),\;i=1,2,\ldots ,n$ is given. Here, $y_{i}$ are identically distributed as $y_{i\;}∼\;W\left(\gamma ,\lambda (x_{i})\right),i=1,2,\ldots ,n$, where $\lambda (x_{i})$ is defined by Eq. (7), $x_{i}={\left[\begin{array}{ccc} 1 & x_{i1} & \begin{array}{cc} \cdots & x_{ip} \end{array} \end{array}\right]}^{T}$, and $\delta_{i}$ is defined by Eq. (14). The probability that the i-th individual experiences the event at $y=y_{i}$ is $P\left(y_{i}|\delta_{i}=1\right)=f(y_{i}$,$x_{i})$, *and* the probability that the i-th individual survives up to $y=y_{i}$ *is* $P\left(y_{i}|\delta_{i}=0\right)=S(y_{i}$,$x_{i})$, *with* $f(y_{i}$*,*$x_{i})$ *and* $S(y_{i}$,$x_{i})$ *given by* Eqs. (12) *and (*8*), respectively. Referring to* Eqs. (5) *and (*14*), the likelihood function is defined as*

(15) $$L\left(\varphi \right)=\prod_{i=1}^{n}f{\left(y_{i},x_{i}\right)}^{\delta_{i\;}}{\left(S\left(y_{i},x_{i}\right)\right)}^{1-\delta_{i}}=\prod_{i=1}^{n}h{\left(y_{i},x_{i}\right)}^{\delta_{i\;}}S\left(y_{i},x_{i}\right),$$

and referring to Eqs. (8), (10), and (14), the likelihood function in (15) can be expressed as:

(16) $$L\left(\varphi \right)=\prod_{i=1}^{n}{\left[\gamma y_{i}^{\gamma -1}\exp\left(b^{T}x_{i}\right)\right]}^{\delta_{i\;}}\exp\left[-y_{i}^{\gamma }\exp\left(b^{T}x_{i}\right)\right].$$

Mathematically, the maximum likelihood (ML) estimator $\hat{\varphi }$ can be obtained by maximizing the log-likelihood function, which is equivalent to maximizing the likelihood function [13,42]. Applying the natural logarithm to the likelihood function in Eq. (16) yields the following log-likelihood function:

(17) $$l\left(\varphi \right)=\log\left(L\left(\varphi \right)\right)=\sum_{i=1}^{n}\left(\delta_{i}\left[\log\gamma +\left(\gamma -1\right)\log y_{i}+b^{T}x_{i}\right]-y_{i}^{\gamma }\exp\left(b^{T}x_{i}\right)\right).$$

In general, the ML estimator $\hat{\varphi }$, which maximizes the log-likelihood function in Eq. (17), can be obtained by taking its first partial derivatives with respect to all parameters and setting them equal to zero [43]. This process yields a nonlinear system of equations, called the log-likelihood equations:

(18) $$\begin{array}{c} \frac{\partial l\left(\varphi \right)}{\partial \varphi }=0, \end{array}$$

where **0** is a zero vector of dimension (p +2). The left-hand side of Eq. (18) is the gradient vector, which has the general form:

(19) $$v\left(\varphi \right)=\frac{\partial l\left(\varphi \right)}{\partial \varphi }={\left[\begin{array}{cc} \frac{\partial l\left(\varphi \right)}{\partial \gamma } & \frac{\partial l\left(\varphi \right)}{\partial b^{T}} \end{array}\right]}^{T}.$$

The components of the gradient vector in Eq. (19) are given by:

(20) $$\frac{\partial l\left(\varphi \right)}{\partial \gamma }=\sum_{i=1}^{n}\left(\delta_{i}\left[\frac{1}{\gamma }+\log y_{i}\right]-y_{i}^{\gamma }\left(\log y_{i}\right)\exp\left(b^{T}x_{i}\right)\right),$$

and

(21) $$\frac{\partial l\left(\varphi \right)}{\partial b^{T}}=\sum_{i=1}^{n}\left(\delta_{i}-y_{i}^{\gamma }\exp\left(b^{T}x_{i}\right)\right)x_{i}^{T},$$

*where* $x_{i}^{T}=\;\left[\begin{array}{ccc} x_{i0} & x_{i1} & \begin{array}{ccc} \ldots & x_{ik} & \begin{array}{cc} \ldots & x_{ip} \end{array} \end{array} \end{array}\right]$, with $x_{i0}=1$. *The partial derivative of the log-likelihood function given in* Eq. (17) *with respect to* $b_{k}$ *is given by*

$$\frac{\partial l\left(\varphi \right)}{\partial b_{k}}=\sum_{i=1}^{n}\left(\delta_{i}-y_{i}^{\gamma }\exp\left(b^{T}x_{i}\right)\right)x_{ik}.$$

*for k* =0,1,2,…,p. *Referring to* Eqs. (17)*, (*20*), and (*21*), the log-likelihood equation given by* Eq. (18) *is nonlinear and cannot be solved analytically to obtain a closed-form solution for the ML estimator. Therefore, the ML estimator is approximated numerically, and one of the commonly used numerical methods is the iterative Newton-Raphson algorithm. The iterative Newton-Raphson procedure is defined as*

(22) $$\begin{array}{c} {\hat{\varphi }}^{\left(m+1\right)}={\hat{\varphi }}^{\left(m\right)}-{\left[H\left({\hat{\varphi }}^{\left(m\right)}\right)\right]}^{-1}v{\left(\hat{\varphi }\right)}^{\left(m\right)},\;m=0,1,2,\cdots . \end{array}$$

*where* $v{\left(\hat{\varphi }\right)}^{\left(m\right)}$ *is the estimated gradient vector given by* Eq. (19) *at the m-th* iteration, and $H({\hat{\varphi }}^{\left(m\right)})$ *is the estimated Hessian matrix based on the data at the m-th iteration. The Hessian matrix is symmetric and represents the matrix of second-order partial derivatives of the log-likelihood function given by* Eq. (17) *with respect to all combinations of the components of the vector* φ*, and it has the general form:*

(23) $$H\left(\varphi \right)=\frac{\partial^{2}l\left(\varphi \right)}{\partial \varphi \partial \varphi^{T}}={\left[\begin{array}{cc} \frac{\partial^{2}l\left(\varphi \right)}{\partial \gamma^{2}} & \frac{\partial^{2}l\left(\varphi \right)}{\partial \gamma \partial b^{T}}\\ \frac{\partial^{2}l\left(\varphi \right)}{\partial b\partial \gamma } & \frac{\partial^{2}l\left(\varphi \right)}{\partial b\partial b^{T}} \end{array}\right]}_{\left(p+2\right)x\left(p+2\right)}.$$

The elements of the Hessian matrix given in Eq. (23) are as follows. Based on Eq. (20), it is obtained

(24) $$\frac{\partial^{2}l\left(\varphi \right)}{\partial \gamma^{2}}=-\sum_{i=1}^{n}\left(\frac{\delta_{i}}{\gamma^{2}}+y_{i}^{\gamma }{\left(\log y_{i}\right)}^{2}\exp\left(b^{T}x_{i}\right)\right),$$

and

(25) $$\frac{\partial^{2}l\left(\varphi \right)}{\partial b^{T}\partial \gamma }=\frac{\partial }{\partial b^{T}}\left(\frac{\partial l\left(\varphi \right)}{\partial \gamma }\right)=-\sum_{i=1}^{n}\left(y_{i}^{\gamma }\left(\log y_{i}\right)\exp\left(b^{T}x_{i}\right)\right)x_{i}^{T}.$$

Referring to Eq. (21), it is obtained

(26) $$\frac{\partial^{2}l\left(\varphi \right)}{\partial b\partial b^{T}}=\frac{\partial }{\partial b}\left(\frac{\partial l\left(\varphi \right)}{\partial b^{T}}\right)=-\sum_{i=1}^{n}\left(y_{i}^{\gamma }\exp\left(b^{T}x_{i}\right)\right)x_{i}x_{i}^{T},$$

with

$$\frac{\partial^{2}l\left(\varphi \right)}{\partial b_{k}\partial b_{l}}=\frac{\partial }{\partial b_{k}}\left(\frac{\partial l\left(\varphi \right)}{\partial b_{l}}\right)=-\sum_{i=1}^{n}\left(y_{i}^{\gamma }\exp\left(b^{T}x_{i}\right)\right)x_{ik}x_{il};\;k,\;l=0,\;1,\;\ldots ,\;p.$$

Based on the Hessian Matrix given in Eq. (23), the Fisher information matrix can be expressed as

(27) $$I_{F}\left(\varphi \right)=-E\left(\frac{\partial^{2}l\left(\varphi \right)}{\partial \varphi \partial \varphi^{T}}\right)=-E\left(H\left(\varphi \right)\right),$$

where E(.) denotes the expectation operator. The variance of the ML estimator, $\text{var}(\hat{\varphi })$, referring to Eq. (27), is given by

(28) $$\begin{array}{c} \text{var}\left(\hat{\varphi }\right)={\left[I_{F}\left(\hat{\varphi }\right)\right]}^{-1}=-{\left[H\left(\hat{\varphi }\right)\right]}^{-1}. \end{array}$$

### Hypothesis testing of parameters in the Weibull regression model

The regression parameter testing of the WR model consists of simultaneous and partial tests. The simultaneous parameter test is used to evaluate the adequacy or goodness of fit of the WR model [14]. The hypotheses for the simultaneous regression parameter test are formulated as follows:

$H_{0}:\;b_{1}=b_{2}=\ldots =b_{p}=0$ (*the WR model is not fit*)

(29) $$H_{a}:\;At\;least\;one\;b_{k}\neq 0,k=1,2,\;\ldots ,\;p\;\left(the\;WR\;model\;is\;fit\right)$$

The test statistic is the Wilks’ likelihood ratio statistic, defined as

(30) $$\begin{array}{c} W=2\left(l\left(\hat{\varphi }\right)-l\left({\hat{\varphi }}_{0}\right)\right), \end{array}$$

*where* $l(\hat{\varphi })$ *is the maximum value of the log-likelihood function given in* Eq. (17)*, and* $l({\hat{\varphi }}_{0})$ *is the maximum value of the log-likelihood function of the WR model under the null hypothesis (H_0_), expressed as*

(31) $$\begin{array}{c} l\left({\hat{\varphi }}_{0}\right)=\sum_{i=1}^{n}\left(\delta_{i}\left[\log{\hat{\gamma }}_{0}+\left({\hat{\gamma }}_{0}-1\right)\log y_{i}+{\hat{b}}_{00}\right]-y_{i}^{\gamma }\exp\left({\hat{b}}_{00}\right)\right), \end{array}$$

where ${\hat{\varphi }}_{0}={\left[\begin{array}{cc} {\hat{\gamma }}_{0} & {\hat{b}}_{00} \end{array}\right]}^{T}$ is the estimated parameter vector of the WR model under H_0_. Under null hypothesis, the test statistic W given in Eq. (30) asymptotically follows a Chi-square distribution with p degrees of freedom, i.e., $W\;∼{\;\chi }_{p}^{2}.$ Test statistic in Eq. (30) can also be approximated by

(32) $$W\approx {\hat{B}}^{T}{\left[I_{F}^{22}\right]}^{-1}\hat{B},$$

*where* $\hat{B}={\left[\begin{array}{ccc} {\hat{b}}_{1} & {\hat{b}}_{2} & \begin{array}{cc} \cdots & {\hat{b}}_{p} \end{array} \end{array}\right]}^{T}$, *and* $\left[I_{F}^{22}\right]$ *is obtained from the variance matrix given in* Eq. (28) *by removing the first and second rows and the first and second columns. The critical region for testing* $H_{0}$ *defined in* Eq. (29)*, is given as follows: H_0_ is rejected at the significance level* α *if and only if* $W\geq \chi_{\left(1-\alpha ;\;p\right)}^{2}$, *or equivalently, if and only if* p−value<α*, with*

(33) p−value=1−F(W),

*where F being the CDF of the chi-squared distribution with* p *degrees of freedom, and* $\chi_{\left(1-\alpha ,\;p\right)}^{2}$ *is a critical value such that* $F(\chi_{\left(1-\alpha ,\;p\right)}^{2})=1-\alpha$ [14,34,44].

The second regression parameter test is the partial test. The hypothesis for the partial test of $b_{k},$ for a given k (k=1,2,…,p), is formulated as follows:

(34) $$H_{0}:\;b_{k}=0\;versus\;H_{a}:\;b_{k}\neq 0\;.$$

The test statistic is Wald statistic, defined as

(35) $$Q_{k}=\frac{{\hat{b}}_{k}}{\sqrt{\text{var}\left({\hat{b}}_{k}\right)}},$$

*where* $Q_{k}\overset{d}{\to }Z\;$ *as* n→∞*, with* Z∼N(0,1)*, and* $\text{var}({\hat{b}}_{k})$ *is the (k + 1)-th element on the main diagonal of* $\text{var}(\hat{\varphi })$ *given in* Eq. (28)*. The null hypothesis in* Eq. (34) *is rejected at the significance level* α *if and only if* $|Q_{k}|>\;Z_{1-\alpha /2}$, *or equivalently, if and only if* p−value<α, *where* $Z_{1-\alpha /2}$ *is the critical value satisfying* $F_{N}\left(Z_{1-\alpha /2}\right)=1-\alpha /2$, *and*

(36) $$p-value=2\left(1-F_{N}\left(\left|Q_{k}\right|\right)\right),$$

with F_N_ being the CDF of the standard normal distribution.

### Geographically weighted Weibull regression model

The geographically weighted Weibull regression (GWWR) is a local form of the Weibull regression (WR) model in which spatial effects are incorporated [34,45,46]. The parameters of the GWWR model are estimated at each observation location using spatial weighting, so that the estimated parameters depend on the geographical position of each observation and vary across locations [47,48]. Suppose the coordinates of each observation point are known and represented by the vector $u_{i}={\left[\begin{array}{cc} u_{i} & v_{i} \end{array}\right]}^{T}$, where $u_{i}$ and $v_{i}$ denote the latitude and longitude of the i-th observation point, respectively. As a local version of the WR model, the GWWR is formulated based on the Weibull distribution, with the scale parameter defined as a function of regression parameters that vary with geographical location. Referring to Eq. (7), the scale parameter given in Eq. (2) can therefore be expressed as a function of the local regression parameters at location $u_{i}$, as follows:

(37) $$\lambda \left(x_{i}\right)=\text{exp}\left(b^{T}\left(u_{i}\right)x_{i}\right)=\text{exp}\left(b_{0}\left(u_{i}\right)+b_{1}\left(u_{i}\right)x_{i1}+b_{2}\left(u_{i}\right)x_{i2}+\cdots +b_{p}\left(u_{i}\right)x_{ip}\right),$$

where $b^{T}\left(u_{i}\right)=\left[\begin{array}{ccc} b_{0}\left(u_{i}\right) & b_{1}\left(u_{i}\right) & \begin{array}{cc} \cdots & b_{p}\left(u_{i}\right) \end{array} \end{array}\right]$ and $x_{i}={\left[\begin{array}{ccc} 1 & x_{i1} & \begin{array}{cc} x_{i2} & \begin{array}{cc} \cdots & x_{ip} \end{array} \end{array} \end{array}\right]}^{T}$. Referring to Eqs. (8) and (37), the spatial Weibull survival regression model at the observation point $u_{i}$ can be obtained, as follows:

(38) $$S\left(y_{i},x_{i},\varphi \left(u_{i}\right)\right)=S\left(y_{i},x_{i}\right)=P(Y>y_{i}|x_{i})=\text{exp}\left[-\;y_{i}^{\gamma \left(u_{i}\right)}\text{exp}\left(b^{T}\left(u_{i}\right)x_{i}\right)\right],$$

where $\varphi \left(u_{i}\right)={\left[\begin{array}{cc} \gamma (u_{i}) & b^{T}\left(u_{i}\right) \end{array}\right]}^{T}={\left[\begin{array}{ccc} \gamma (u_{i}) & b_{0}\left(u_{i}\right) & \begin{array}{ccc} b_{1}\left(u_{i}\right) & \cdots & b_{p} \end{array} \end{array}\left(u_{i}\right)\right]}^{T}$. Similarly, referring to Eqs. (9) and (37), the spatial Weibull regression model for the cumulative distribution function at the observation point ***u_i_*** can be formulated as follows:

(39) $$F\left(y_{i},x_{i},\varphi \left(u_{i}\right)\right)=F\left(y_{i},x_{i}\right)=P(Y\leq y_{i}|x_{i})=1-\text{exp}\left[-\;y_{i}^{\gamma \left(u_{i}\right)}\text{exp}\left(b^{T}\left(u_{i}\right)x_{i}\right)\right].$$

Referring to Eqs. (10) and (37), the spatial Weibull hazard regression model at location ***u_i_*** can be expressed as:

(40) $$h\left(y_{i},x_{i},\varphi \left(u_{i}\right)\right)=h\left(y_{i},x_{i}\right)=\gamma \left(u_{i}\right)y_{i}^{\gamma \left(u_{i}\right)-1}\;\text{exp}\left(b^{T}\left(u_{i}\right)x_{i}\right),$$

and referring to Eqs. (11) and (37), the spatial Weibull mean regression model at location ***u_i_*** can be formulated as:

(41) $$\mu_{Y}\left(x_{i},b\left(u_{i}\right)\right)=\mu \left(x_{i}\right)=\Gamma \left(1+\frac{1}{\gamma \left(u_{i}\right)}\right)\text{exp}\left(-\frac{1}{\gamma }b^{T}\left(u_{i}\right)x_{i}\right).$$

Based on Eqs. (12) and (37), the PDF at location ***u_i_***, which depends on the local regression parameters, can be expressed as follows:

(42) $$f\left(y_{i},x_{i},\;\varphi \left(u_{i}\right)\right)=f\left(y_{i},x_{i}\right)=\gamma \left(u_{i}\right)y_{i}^{\gamma \left(u_{i}\right)-1}\left[\text{exp}\left(b^{T}\left(u_{i}\right)x_{i}\right)\right]\text{exp}\left[-y_{i}^{\gamma \left(u_{i}\right)}\text{exp}\left(b^{T}\left(u_{i}\right)x_{i}\right)\right].$$

It is known that the WR model given in Eqs. (8)-(12) is considered the global version of the GWWR model, that is, a GWWR model in which the parameter values are identical across all observation locations.

The spatial effect in the GWWR model is implemented by incorporating spatial weights into the parameter estimation process. Spatial weights are calculated using a spatial weighting function, with one commonly used function being the adaptive Gaussian function. Let $w_{ij}$ denotes the spatial weight assigned to the data at the j-th observation point for estimating the parameters of the GWWR model at the i-th observation point, located at coordinate $u_{i}$. Using the adaptive Gaussian function, the spatial weights are calculated as follows:

(43) $$w_{ij}=\text{exp}\left[-\frac{1}{2}{\left(\frac{d_{ij}}{a_{i}}\right)}^{2}\right],j=1,2,\;\ldots ,\;n,$$

where $d_{ij}$ is the Euclidean distance between the observation at points ***u_i_*** and $u_{j},$ $a_{i}$ is the adaptive bandwidth for parameter estimation of GWWR model at observation point ***u_i_***, and its value differs across observation locations. Another commonly used spatial weighting function is the adaptive bi-square function, defined as:

(44) $$w_{ij}=\;\left\{ \begin{array}{c} {\left[1-{\left(\frac{d_{ij}}{a_{i}}\right)}^{2}\right]}^{2},\;if\;d_{ij}\leq a_{ij}\\ 0,\;if\;d_{ij}>a_{ij} \end{array}\right.;j=\;1,2,\;\ldots ,n\;.$$

Determining the optimal bandwidth is a critical issue in estimating the parameters of the GWR model. One approach to determine the optimal bandwidth is based on the Bayesian Information Criterion (BIC). The optimal bandwidth is defined as the value that minimizes the BIC of the GWWR model. The BIC is calculated using the following formula:

(45) $$\begin{array}{c} \mathrm{BIC}\left(u_{i}\right)=-2l\left(\hat{\varphi }\left(u_{i}\right)\right)+K.\log\left(n\right), \end{array}$$

where $l(\hat{\varphi }\left(u_{i}\right))$ is the maximum of the log-likelihood function, K is the number of model parameters, and n is the sample size. In addition, the model goodness-of-fit measures used in this study are the generalized cross-validation (GCV), the McFadden $R^{2},$ and the mean absolute percentage error (MAPE).

### Parameter estimation in the geographically weighted Weibull regression model

The proposed parameter estimation method for the GWWR model in this study is MLE with spatial weighting and data censoring. This approach combines the parameter estimation method of the GWR model with the adaptation of the Weibull regression model for right-censored time-to-event data [14,49,50]. The MLE method with spatial weighting is commonly used to estimate the parameters of nonlinear GWR models, including the GWWR model*. Referring to* Eq. (15)*,* the local likelihood function incorporating spatial weighting and data censoring for parameter estimation of the GWWR model at observation location $u_{i}$ is defined as:

(46) $$L\left(\varphi \left(u_{i}\right)\right)=\prod_{j=1}^{n}{\left[f{\left(y_{j},x_{j}\right)}^{\delta_{j\;}}{\left(S\left(y_{j},x_{j}\right)\right)}^{1-\delta_{j}}\right]}^{w_{ij}}=\prod_{j=1}^{n}{\left[h{\left(y_{j},x_{j}\right)}^{\delta_{j}}S\left(y_{j},x_{j}\right)\right]}^{w_{ij}},$$

where $\delta_{j}$ denotes the censoring status of the response variable at the j-th observation point, as defined in Eq. (14), and $w_{ij}$ is the spatial weight calculated using Eq. (43) or (44). Mathematically, the ML estimator of the GWWR model can be more easily obtained by maximizing the log-likelihood function, since the maxima of both the likelihood function and its log-likelihood function occur at the same point, namely, $\hat{\varphi }\left(u_{i}\right)$ (the ML estimator). Applying the natural logarithm to both sides of Eq. (46) yields the local log-likelihood function incorporating spatial weights and data censoring:

(47) $$\ell \left(\varphi \left(u_{i}\right)\right)=\log\left(L\left(\varphi \left(u_{i}\right)\right)\right)=\sum_{j=1}^{n}w_{ij}\left(\delta_{j}\text{log}\left[h\left(y_{j},x_{j}\right)\right]+\text{log}\left[S\left(y_{j},x_{j}\right)\right]\right).$$

Referring to Eqs. (38) and (40), the expression of the log-likelihood function in Eq. (47) can be expressed a:

(48) $$l\left(\varphi \left(u_{i}\right)\right)=\sum_{j=1}^{n}w_{ij}\left(\delta_{j}\left[\log\gamma \left(u_{i}\right)+\left(\gamma \left(u_{i}\right)-1\right)\log y_{j}+b^{T}\left(u_{i}\right)x_{j}\right]-y_{j}^{\gamma \left(u_{i}\right)}\exp\left(b^{T}\left(u_{i}\right)x_{j}\right)\right).$$

The ML estimator of the GWWR model at observation location $u_{i}$ is obtained by solving the corresponding log-likelihood equation:

(49) $$\begin{array}{c} \frac{\partial l\left(\varphi \left(u_{i}\right)\right)}{\partial \varphi \left(u_{i}\right)}=0, \end{array}$$

where **0** on the right-hand side of Eq. (49) denotes a zero vector of dimension p + 2. The term on the left-hand side of Eq. (49) represents the gradient vector for parameter estimation of the GWWR model at i-th observation point and is expressed in the general form:

(50) $$\frac{\partial l\left(\varphi \left(u_{i}\right)\right)}{\partial \varphi \left(u_{i}\right)}=v\left(\varphi \left(u_{i}\right)\right)={\left[\begin{array}{cc} \frac{\partial l\left(\varphi \left(u_{i}\right)\right)}{\partial \gamma \left(u_{i}\right)} & \frac{\partial l\left(\varphi \left(u_{i}\right)\right)}{\partial b^{T}\left(\varphi \left(u_{i}\right)\right)} \end{array}\right]}^{T}.$$

The components of the gradient vector in Eq. (50) are given by:

(51) $$\frac{\partial l\left(\varphi \left(u_{i}\right)\right)}{\partial \gamma \left(u_{i}\right)}=\sum_{j=1}^{n}w_{ij}\left(\delta_{j}\left[\frac{1}{\gamma \left(u_{i}\right)}+\log y_{j}\right]-y_{j}^{\gamma \left(u_{i}\right)}\left(\log y_{j}\right)\exp\left(b^{T}\left(u_{i}\right)x_{j}\right)\right),$$

and

(52) $$\frac{\partial l\left(\varphi \left(u_{i}\right)\right)}{\partial b^{T}\left(u_{i}\right)}=\sum_{j=1}^{n}w_{ij}\left(\delta_{j}-y_{j}^{\gamma \left(u_{i}\right)}\exp\left(b^{T}\left(u_{i}\right)x_{j}\right)\right)x_{j}^{T},\;$$

where $x_{j}=\;\left[\begin{array}{ccc} x_{j0} & x_{j1} & \begin{array}{ccc} \ldots & x_{jk} & \begin{array}{cc} \ldots & x_{jp} \end{array} \end{array} \end{array}\right]{,}^{T}x_{j0}=1,$ representing the vector of covariate data at the j-th observation point. Referring to Eq. (52), for k=0,1,2,…,p, the component of the gradient vector with respect to $b_{k}\left(u_{i}\right)$ is obtained as:

$$\frac{\partial l\left(\varphi \left(u_{i}\right)\right)}{\partial b_{k}\left(u_{i}\right)}=\sum_{j=1}^{n}w_{\mathrm{ij}}\left(\delta_{j}-y_{j}^{\gamma \left(u_{i}\right)}\exp\left(b^{T}\left(u_{i}\right)x_{j}\right)\right)x_{\mathrm{jk}}.$$

Based on the expressions in Eqs. (51) and (52), the log-likelihood Eq. (49) is a nonlinear system of equations and therefore cannot be solved analytically to obtain a closed-form expression for the ML estimator. Hence, a numerical method is required to estimate the parameters. In this study, the Newton–Raphson iterative method is proposed. The Newton–Raphson iterative algorithm is given by:

(53) $$\begin{array}{c} \hat{\varphi }{\left(u_{i}\right)}^{\left(q+1\right)}=\hat{\varphi }{\left(u_{i}\right)}^{\left(q\right)}-{\left[H{\left(\hat{\varphi }\left(u_{i}\right)\right)}^{\left(q\right)}\right]}^{-1}\;v\left({\left(\hat{\varphi }\left(u_{i}\right)\right)}^{\left(q\right)}\right),\;q=0,\;1,\;2,\;\cdots ., \end{array}$$

where $v(\varphi (u_{i}))$ denotes the gradient vector given in Eq. (50), and $H(\varphi (u_{i}))$ is the Hessian matrix used for parameter estimation of the GWWR model at the i-th observation location. Referring to Eq. (53), the general form of the Hessian matrix is given as follows:

(54) $$H\left(\varphi \left(u_{i}\right)\right)=\frac{\partial^{2}l\left(\varphi \left(u_{i}\right)\right)}{\partial \varphi \left(u_{i}\right)\partial \varphi^{T}\left(u_{i}\right)}={\left[\begin{array}{cc} \frac{\partial^{2}l\left(\varphi \left(u_{i}\right)\right)}{\partial \gamma^{2}\left(u_{i}\right)} & \frac{\partial^{2}l\left(\varphi \left(u_{i}\right)\right)}{\partial \gamma \left(u_{i}\right)\partial b^{T}\left(u_{i}\right)}\\ \frac{\partial^{2}l\left(\varphi \left(u_{i}\right)\right)}{\partial b\left(u_{i}\right)\partial \gamma \left(u_{i}\right)} & \frac{\partial^{2}l\left(\varphi \left(u_{i}\right)\right)}{\partial b\left(u_{i}\right)\partial b^{T}\left(u_{i}\right)} \end{array}\right]}_{\left(p+2\right)x\left(p+2\right)}.$$

The elements of the Hessian matrix given in Eq. (54) are computed as follows. By differentiating Eq. (51) with respect to the parameter $\gamma (u_{i})$ and the vector $b(u_{i}),$ respectively, it is obtained

(55) $$\frac{\partial^{2}l\left(\varphi \left(u_{i}\right)\right)}{\partial \gamma^{2}\left(u_{i}\right)}=-\sum_{j=1}^{n}w_{ij}\left(\frac{\delta_{j}}{\gamma^{2}\left(u_{i}\right)}+y_{j}^{\gamma \left(u_{i}\right)}{\left(\log y_{j}\right)}^{2}\exp\left(b^{T}\left(u_{i}\right)x_{j}\right)\right),$$

and

(56) $$\frac{\partial^{2}l\left(\varphi \left(u_{i}\right)\right)}{\partial b\left(u_{i}\right)\partial \gamma \left(u_{i}\right)}={\left[\frac{\partial^{2}l\left(\varphi \left(u_{i}\right)\right)}{\partial \gamma \left(u_{i}\right)\partial b^{T}\left(u_{i}\right)}\right]}^{T},$$

with

$$\frac{\partial^{2}l\left(\varphi \left(u_{i}\right)\right)}{\partial \gamma \left(u_{i}\right)\partial b^{T}\left(u_{i}\right)}=\frac{\partial }{\partial \gamma \left(u_{i}\right))}\left(\frac{\partial l\left(\varphi \left(u_{i}\right)\right)}{\partial \gamma \left(u_{i}\right)b^{T}\left(u_{i}\right)}\right)=-\sum_{j=1}^{n}w_{ij}\left(y_{j}^{\gamma \left(u_{i}\right)}\left(\log y_{j}\right)\exp\left(b^{T}\left(u_{i}\right)x_{j}\right)\right)x_{j}^{T}.$$

Referring to Eq. (52), the following expression is obtained:

(57) $$\frac{\partial^{2}l\left(\varphi \left(u_{i}\right)\right)}{\partial b\left(u_{i}\right)\partial b^{T}\left(u_{i}\right)}=\frac{\partial }{\partial b\left(u_{i}\right)}\left(\frac{\partial l\left(\varphi \left(u_{i}\right)\right)}{\partial b^{T}\left(u_{i}\right)}\right)=-\sum_{j=1}^{n}w_{ij}\left(y_{j}^{\gamma \left(u_{i}\right)}\exp\left(b^{T}\left(u_{i}\right)x_{j}\right)\right)x_{j}x_{j}^{T},$$

and for k,l=0,1,2,…,p,

$$\frac{\partial^{2}l\left(\varphi \left(u_{i}\right)\right)}{\partial b_{k}\left(u_{i}\right)\partial b_{l}\left(u_{i}\right)}=\frac{\partial }{\partial b_{k}\left(u_{i}\right)}\left(\frac{\partial l\left(\varphi \left(u_{i}\right)\right)}{\partial b_{l}\left(u_{i}\right)}\right)=-\sum_{j=1}^{n}w_{ij}\left(y_{j}^{\gamma \left(u_{i}\right)}\exp\left(b^{T}\left(u_{i}\right)x_{j}\right)\right)x_{jk}x_{jl}.$$

Based on the Hessian matrix given in Eq. (54), the variance–covariance matrix of the ML estimator $\hat{\varphi }\left(u_{i}\right)$ can be obtained as:

(58) $$\begin{array}{c} \text{var}\left(\hat{\varphi }\left(u_{i}\right)\right)={\left[I_{F}\left(\hat{\varphi }\left(u_{i}\right)\right)\right]}^{-1}=-{\left[H\left(\hat{\varphi }\left(u_{i}\right)\right)\right]}^{-1}, \end{array}$$

where $\left[I_{F}\left(\hat{\varphi }\left(u_{i}\right)\right)\right]$ denotes the Fisher information matrix corresponding to the parameter estimation of the GWWR model at the i-th observation location point, defined as:

$$I_{F}\left(\varphi \left(u_{i}\right)\right)=-E\left[\frac{\partial^{2}l\left(\varphi \left(u_{i}\right)\right)}{\partial \varphi \left(u_{i}\right)\partial \varphi^{T}\left(u_{i}\right)}\right]=-E\left[H\left(\varphi \left(u_{i}\right)\right)\right].$$

When the ML estimator of the GWWR model yields identical values at all observation locations, the model simplifies to a global form, known as the WR model. Accordingly, the ML estimator of the WR model can be obtained by estimating the parameters of the GWWR model with an identity spatial weight matrix, indicating that a weight of one is assigned to each observation point [4,34].

### Parameter testing of the geographically weighted Weibull regression model

Parameter testing of the GWWR model consists of three stages: the similarity test between the GWWR model and the global model, the simultaneous parameter test, and the partial parameter test. The similarity test is performed to assess whether the GWWR model provides a significantly better fit to the data than the global WR model. The hypotheses for the similarity test are defined as follows:

$H_{0}:\;b_{k}\left(u_{i}\right)=b_{k};\;k=1,\;2,\;\ldots ,\;p;\;i=1,\;2,\;\ldots ,\;n$ (the fitted model is the WR, i.e., the global model),

$H_{a}:At\;least\;one\;b_{k}\left(u_{i}\right)\neq \beta_{k},for\;k=1,\;2,\;\ldots ,\;p,\;and\;i=1,\;2,\;\ldots ,\;n$ (the fitted model is the GWWR).

The test statistic is developed based on Wilks’ likelihood ratio, which is defined as:

(59) $$\begin{array}{c} G=2\sum_{i=1}^{n}\left(l\left(\hat{\varphi }\left(u_{i}\right)\right)-l\left(\hat{\varphi }\right)\right), \end{array}$$

with

$$l\left(\hat{\varphi }\right)=\sum_{i=1}^{n}\left(\delta_{i}\left[\log\hat{\gamma }+\left(\hat{\gamma }-1\right)\log y_{i}+{\hat{b}}^{T}x_{i}\right]-y_{i}^{\hat{\gamma }}\exp\left({\hat{b}}^{T}x_{i}\right)\right),$$

represents the maximum value of the log-likelihood function for parameter estimation of the WR model, as given in Eq. (17). Similarly,

(60) $$\begin{array}{c} l\left(\hat{\varphi }\left(u_{i}\right)\right)=\sum_{j=1}^{n}w_{\mathrm{ij}}\left(\delta_{j}\left[\log\hat{\gamma }\left(u_{i}\right)+\left(\hat{\gamma }\left(u_{i}\right)-1\right)\log y_{j}+{\hat{b}}^{T}\left(u_{i}\right)x_{j}\right]-y_{j}^{\hat{\gamma }}\left(u_{i}\right)\exp\left({\hat{b}}^{T}\left(u_{i}\right)x_{j}\right)\right), \end{array}$$

denotes the maximum value of the log-likelihood function for parameter estimation of the GWWR model at the i-th observation point, as given in Eq. (48). It follows from Eq. (59) that each component $2(l\left(\hat{\varphi }\left(u_{i}\right)\right)-l\left(\hat{\varphi }\right))$ asymptotically follows a chi-square distribution with p degrees of freedom, i.e., $\chi_{\left(p\right)}^{2}$. Consequently, the overall test statistic G given by Eq. (59) follows a chi-square distribution with np degrees of freedom, $\chi_{\left(np\right)}^{2}$. The null hypothesis is rejected at the significance level α if and only if $G>\chi_{\left(1-\alpha ;\;np\right)}^{2}$. Equivalently, $H_{0}$ is rejected if and only if the p−value<α, where p−value=1−F(G), and F(G) denotes the CDF of the chi-square distribution with np degrees of freedom evaluated at G. Referring to Eq. (32), the test statistic G given in Eq. (59) can be approximated as

(61) $$G\approx \sum_{i=1}^{n}C\left(u_{i}\right),$$

where $C\left(u_{i}\right)={\left[\hat{B}\left(u_{i}\right)-\hat{B}\right]}^{T}{\left[I_{F}^{22}\left(\varphi (u_{i})\right)\right]}^{-1}\left[\hat{B}\left(u_{i}\right)-\hat{B}\right]\;∼\chi_{\left(p\right)}^{2}$. Here $\hat{B}\left(u_{i}\right)={\left[\begin{array}{ccc} {\hat{b}}_{1}\left(u_{i}\right) & {\hat{b}}_{2}\left(u_{i}\right) & \begin{array}{cc} \cdots & {\hat{b}}_{p}\left(u_{i}\right) \end{array} \end{array}\right]}^{T},$ and the vector $\hat{B}$ is defined in Eq. (32). The matrix $I_{F}^{22}\left(\varphi (u_{i})\right)$ can be obtained from the variance-covariance matrix given in Eq. (58) by removing the first and second rows, as well as the first and second columns [51,52].

The second stage of the GWWR model evaluation involves simultaneous parameter testing. This test aims to confirm whether the GWWR model adequately represents a local WR model, that is, whether at least one local regression parameter is significantly different from zero. The hypotheses of this test are formulated as follows:

$H_{0}:\;b_{1}\left(u_{i}\right)=b\left(u_{i}\right)=\ldots =b\left(u_{i}\right)=0$; i=1,2,…,n (no local model fit),

$H_{a}:at\;least\;there\;is\;one\;b\left(u_{i}\right)\neq 0;k=1,2,\;\ldots ,p;i=1,2,\;\ldots ,\;n$

(presence of a local model, i.e., the GWWR model)

The test statistic is defined as:

(62) $$\begin{array}{c} G_{w}=2\sum_{i=1}^{n}\left(l\left(\hat{\varphi }\left(u_{i}\right)\right)-l\left({\hat{\varphi }}_{0}\left(u_{i}\right)\right)\right), \end{array}$$

where $l(\hat{\varphi }\left(u_{i}\right))$ is given by Eq. (60), and $l({\hat{\varphi }}_{0}\left(u_{i}\right))$ denotes the maximum value of the log-likelihood function for parameter estimation of GWWR model at the i-th observation point under the null hypothesis, which is expressed as:

(63) $$\begin{array}{c} l\left({\hat{\varphi }}_{0}\left(u_{i}\right)\right)=\sum_{j=1}^{n}w_{\mathrm{ij}}\left(\delta_{j}\left[\log{\hat{\gamma }}_{0}\left(u_{i}\right)+\left({\hat{\gamma }}_{0}\left(u_{i}\right)-1\right)\log y_{j}+{\hat{b}}_{00}\left(u_{i}\right)\right]-y_{j}^{\hat{\gamma }}\left(u_{i}\right)\exp\left({\hat{b}}_{00}(u_{i}\right)\right). \end{array}$$

Referring to Eq. (30) and under the null hypothesis, the statistic $2\left(l\left(\hat{\varphi }\left(u_{i}\right)\right)-l\left({\hat{\varphi }}_{0}\left(u_{i}\right)\right)\right)\;∼\chi_{\left(p\right)}^{2},$ and therefore $G_{w}∼\chi_{\left(\text{np}\right)}^{2}.$ The statistic $G_{w}$ given in Eq. (62) can be approximated by:

(64) $$G_{w}\approx \sum_{i=1}^{n}C_{w}\left(u_{i}\right),$$

with $C_{w}\left(u_{i}\right)={\hat{B}}^{T}\left(u_{i}\right){\left[I_{F}^{22}\left(\varphi (u_{i})\right)\right]}^{-1}\hat{B}\left(u_{i}\right)\;∼\chi_{\left(p\right)}^{2}$ for i=1,2,…,n.

The third stage of the GWWR model parameter evaluation is partial parameter testing. The goal of this test is to assess the individual influence of each covariate on the model. For given k and i, with k=1,2,…,p and i=1,2,…,n, the hypotheses for the partial parameter test are formulated as:

$H_{0}:\;b_{k}\left(u_{i}\right)=0$ (*the covariate* $X_{k}$ *does not influence the GWWR model*),

$H_{a}:\;b_{k}\left(u_{i}\right)\neq 0\;$(*the covariate* $X_{k}$ *influences the GWWR model*).

*The test statistic is Wald statistic, which is defined as follows:*

(65) $$W_{k}\left(u_{i}\right)=\frac{{\hat{b}}_{k}\left(u_{i}\right)}{\sqrt{\text{var}\left({\hat{b}}_{k}\left(u_{i}\right)\right)}},$$

*with* $W_{k}\left(u_{i}\right)\overset{d}{\to }Z\;as\;n\to \infty ,\;$ where Z∼N(0,1), *and* $\text{var}({\hat{b}}_{k}\left(u_{i}\right))$ *is the (k+2)-th on the main diagonal of the variance-covariance matrix given in* Eq. (58). $H_{0}$ *is rejected at the significance level* α *If and only if* $|W_{k}\left(u_{i}\right)|>Z_{\left(1-\frac{\alpha }{2}\right)}$, *or equivalently,* $H_{0}$ *is rejected if and only if* p−value<α, *where* $p-value=2\left(1-F_{Z}\left(|W_{k}\left(u_{i}\right)|\right)\right)=P\left(\left|Z\right|\right)>|W_{k}\left(u_{i}\right)\left|\right);Z\;∼\;N\left(0,1\right),$ and $F_{Z}$ is the CDF of the standard normal distribution [53,54].

### Detection of multicollinearity among covariates

Detection of multicollinearity among covariates aims to ensure that parameter estimation can be properly performed and that the GWWR model analysis is appropriate [55]. One common approach for detecting multicollinearity is the Variance Inflation Factor (VIF). A VIF value greater than 10 indicates the presence of multicollinearity among covariates [56,57]. The VIF for the k-th covariate is computed using the following formula:

(66) $$VIF_{k}=\frac{1}{1-R_{k}^{2}},$$

*with* $R_{k}^{2}$ *is the coefficient of determination from the regression of the k-th covariate on the remaining covariates, which can be computed using the following formula:*

(67) $$R_{k}^{2}=1-\frac{\sum_{i=1}^{n}{\left(x_{ki}-{\hat{X}}_{ki}\right)}^{2}}{\sum_{i=1}^{n}{\left(x_{ki}-{\bar{X}}_{k}\right)}^{2}},$$

*where* $x_{ki}$ *denotes the value of the k-th covariate for the i-th observation,* ${\hat{X}}_{ki}$ *is the estimated value of the k-th covariate at the i-th observation point, and* n *represents the sample size.*

### Test for spatial heterogeneity of the response variable

The purpose of the spatial heterogeneity test is to verify whether the response variable exhibits spatial heterogeneity, which is a prerequisite for conducting GWWR modeling. A commonly used approach to test spatial heterogeneity is the Glejser test [58,59]. The Glejser test simultaneously evaluates the parameters of a multiple linear regression model, where the response variable is defined as $|e_{i}|,\;i=1,\;2,\;\ldots ,n,$ with $e_{i}=y_{i}-{\hat{y}}_{i}.$ Here, $y_{i}$ represents the observed value of the response variable at the i-th location, and ${\hat{y}}_{i}$ represents its estimated value. The hypotheses for the spatial heterogeneity test are formulated as follows:

$H_{0}:\;\sigma_{1}^{2}=\sigma_{2}^{2}=\;\cdots =\sigma_{n}^{2}=\sigma^{2}$ (the response variable data are spatially homogeneous),

$H_{a}$: At least one $\sigma_{i}^{2}\neq \sigma^{2},\;i=1,\;2,\;\ldots ,\;n$ (the response variable data are spatial heterogeneous).

The test statistic is defined as follows:

(68) $$\begin{array}{c} F_{g}=\frac{\left({\hat{\beta }}^{T}X^{T}e-n{\bar{e}}^{2}\right)/p}{\left(e^{T}e-{\hat{\beta }}^{T}X^{T}e\right)/\left(n-p-1\right)}, \end{array}$$

with $\hat{\beta }={\left(X^{T}X\right)}^{-1}X^{T}e$ represents the ordinary least squares (OLS) estimator of the auxiliary regression. **X** denotes the matrix of the observed covariate data, $e={\left[\begin{array}{ccc} |e_{1}| & |e_{2}| & \begin{array}{cc} \cdots & |e_{n}| \end{array} \end{array}\right]}^{T}$, and p is the number of covariates. Under the null hypothesis, the test statistic $F_{g}$ follows an F distribution, i.e., $F_{g}∼F_{\left(p,\;n-p-1\right)}.$ $H_{0}$ is rejected at the significance level α if and only if $F_{g}>F_{\left(1-\alpha ,\;(p,\;n-p-1)\right)}$, or equivalently, if and only if the p−value<α.

### Interpretation of the geographically weighted Weibull regression model

*The RW model was a proportional hazards (PH) model, in which the hazard ratio between any two individuals is constant, that is, the hazard functions of any two individuals are constant multiples of one another. Based on the PH model, the interpretation of the GWWR model relies on the ratio of the predicted GWWR values corresponding to a one-unit increase in each covariate. Specifically, the ratio of the predicted values of the spatial Weibull survival regression model at the i-th observation location, corresponding to a one-unit increase in the k-th continuous covariate, is given by*

(69) $$R_{S}\left(X_{k}\right)=\frac{\hat{S}\left(y_{i},x_{i}|x_{ik}+1\right)}{\hat{S}\left(y_{i},x_{i}\right)}=\frac{\exp\left[-y_{i}^{\hat{\gamma }\left(u_{i}\right)}\text{exp}\left({\hat{b}}_{0}\left(u_{i}\right)+{\hat{b}}_{1}\left(u_{i}\right)x_{i1}+\ldots +{\hat{b}}_{k}\left(u_{i}\right)\left(x_{ik}+1\right)+\ldots +{\hat{b}}_{p}\left(u_{i}\right)x_{ip}\right)\right]}{\exp\left[-y_{i}^{\hat{\gamma \;}\left(u_{i}\right)}\exp\left({\hat{b}}_{0}\left(u_{i}\right)+{\hat{b}}_{1}\left(u_{i}\right)x_{i1}+\ldots +{\hat{b}}_{k}\left(u_{i}\right)x_{ik}+\ldots +{\hat{b}}_{p}\left(u_{i}\right)x_{ip}\right)\right]},$$

*where* $S(y_{i},x_{i})$ *denotes the spatial Weibull survival regression model at the i-th observation location, which is defined in* Eq. (38)*. The ratio of the spatial Weibull cumulative distribution regression model, the spatial Weibull hazard regression, and the spatial Weibull regression for the mean at the observation point* $u_{i},$ *corresponding to a one-unit increase in the continuous covariate* $X_{k}$*, is given by:*

(70) $$R_{F}\left(X_{k}\right)\;=\frac{\hat{F}\left(y_{i},x_{i}|x_{ik}+1\right)}{\hat{F}\left(y_{i},x_{i}\right)}=\frac{1-\hat{S}\left(y_{i},x_{i}|x_{ik}+1\right)}{1-\hat{S}\left(y_{i},x_{i}\right)};$$

(71) $$R_{h}\left(X_{k}\right)\;=\frac{\hat{h}\left(y_{i},x_{i}|x_{ik}+1\right)}{\hat{h}\left(y_{i},x_{i}\right)}=\exp\left({\hat{b}}_{k}\left(u_{i}\right)\right);$$

and

(72) $$R_{\mu }\left(X_{k}\right)\;=\frac{{\hat{\mu }}_{Y}\left(y_{i},x_{i}|x_{ik}+1\right)}{{\hat{\mu }}_{Y}\left(y_{i},x_{i}\right)}=\exp\left({\hat{b}}_{k}\left(u_{i}\right)\right),$$

respectively, where $F(y_{i},x_{i})$, $h(y_{i},x_{i})$, and $\mu_{Y}\left(y_{i},x_{i}\right)$ are given in Eqs. (39), (40), and (41), respectively.

## Method validation

### Data description

The research data were secondary data provided by the Environmental Agency of East Kalimantan Province, consisting of 27 observation points. The dataset included the response variable, covariates, and the coordinates of river water observation points collected in East Kalimantan in 2024. The response variable was the dissolved oxygen (DO) level of river water (Y), while the covariates included dissolved iron (Fe, $X_{1}$) total phosphate $\left(X_{2}\right)$, water temperature $\left(X_{3}\right)$, biochemical oxygen demand (BOD, $X_{4}$), nitrate $\left(X_{5}\right)$, ammonia $\left(X_{6}\right)$, oil and grease $\left(X_{7}\right)$, pH (potential of hydrogen, $X_{8}$), total suspended solids (TSS, $X_{9}$), and water color degree $\left(X_{10}\right)$. The measurement units were milligrams per liter (mg/L) or parts per million (ppm). The data were analyzed using the geographically weighted Weibull regression (GWWR) model, and all computations were performed using GNU Octave (open-source software). Descriptive statistics of the data are presented in Table 1.

**Table 1:** Descriptive statistics of the research data.

Variable	Average	Maximum	Minimum	Standard Deviation	Quality Standard
DO (*Y*)	4.8111	7.0000	3.4000	1.0390	≥6*ppm*
Dissolved Fe (*X*_1_)	0.3067	1.0000	0.0300	0.2524	≤0.3*ppm*
Total Phosphate (*X*_2_)	0.0352	0.1000	0.0250	0.0191	≤0.2 ppm
Water Temperature (*X*_3_)	28.7407	27.0000	23.0000	1.4302	dev 3°C (*)
BOD (*X*_4_)	5.6667	8.0000	3.0000	1.5442	≤2*ppm*
Nitrate (*X*_5_)	3.0011	8.0000	0.0300	1.9789	≤10*ppm*
Ammonia (*X*_6_)	0.0981	0.2000	0.0400	0.0441	≤0.1*ppm*
Oil and Fat (*X*_7_)	0.3704	0.4000	0.3000	0.0465	≤1*ppm*
Water pH (*X*_8_)	6.9700	7.8500	6.1000	0.3991	6≤pH≤9
TSS (*X*_9_)	54.1959	172.0000	1.2900	37.9561	≤40*ppm*
Water Color Degree (*X*_10_)	83.2593	185.0000	8.0000	49.0609	≤15*ppm*

⁎ The maximum difference between the water and air temperatures measured at the water surface is 3°C.

**Fig. 1:**
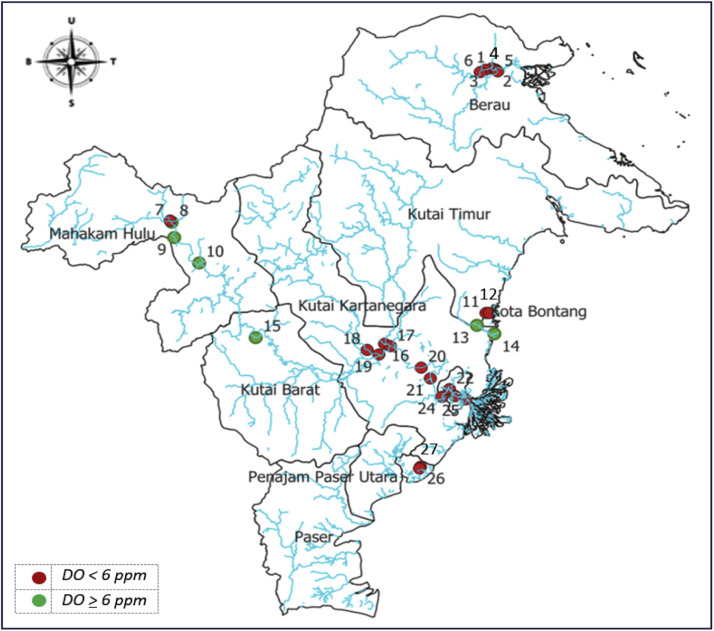
Spatial distribution of river water DO across all observation points in East Kalimantan, 2024.

The average DO value shown in Table 1 indicates that the predicted river water DO in East Kalimantan in 2024 was 4.8111 ppm, with a maximum deviation of 1.0390 ppm. The observed river water DO in ***2024*** was less than 6 ppm, indicating that the river water quality in East Kalimantan is degraded. The lowest DO value was 3.4000 ppm, and the highest was 7.0000 ppm. The spatial distribution of river water DO is visualized in a DO distribution map of all observation points in East Kalimantan (Fig. 1).

Fig. 1 shows a map of East Kalimantan Province, which is divided into ten administrative regions: Samarinda, Balikpapan, Bontang, Paser, Penajam Paser Utara, Kutai Barat, Kutai Kartanegara, Mahakam Hulu, Kutai Timur, and Berau. The blue lines represent rivers distributed across East Kalimantan, while the red and green dots indicate the river water observation locations. The dots in Fig. 1 are numbered to correspond to the names of the observation locations presented in Table 7. The notations U, T, S, and B on the compass points in Fig. 1 represent North, East, South and West, respectively.

The distribution of river water DO, as visualized in Fig. 1, is classified into two categories: less than 6 ppm and greater than or equal to 6 ppm. Red dots represent observation points where river water quality (RWQ) is degraded, indicated by DO levels below 6 ppm, while the green dots denote locations with good water quality. Among the 27 observation points, 21 are marked in red, indicating that 77.78% of the sampled locations in 2024 recorded DO levels below 6 ppm, suggesting that the RWQ at most observation points is degraded.

The average dissolved Fe concentration presented in Table 1 shows that the predicted value was 0.3067 ppm with a maximum deviation of 0.2524 ppm. The dissolved Fe concentration in the river water in East Kalimantan ranged from 0.0300 to 1.0000 ppm, with an average value exceeding the applicable quality standard (≤0.3 ppm). The predicted total phosphate concentration was 0.0352 ppm, with a maximum deviation of 0.0191 ppm. Based on the 2024 field observations, the total phosphate concentration ranged from 0.0250 to 0.1000 ppm, which complies with the quality standard (≤0.2 ppm). The predicted river water temperature in East Kalimantan was $28.7407{\;}^{\circ }C$, with a maximum deviation of $1.4302{\;}^{\circ }C$. Meanwhile, the average air temperature above the water surface was $31.2963{\;}^{\circ }C$, indicating that the river water temperature complies with the quality standard, which requires the difference between the average water and air temperatures to be less than $3{\;}^{\circ }C$. The predicted BOD concentration of river water in East Kalimantan in 2024 was 5.6667 ppm, with a maximum deviation of 1.5442 ppm. The observed BOD concentration ranged from 3.0000 to 8.0000 ppm, indicating that it exceeds the quality standard (≤2 ppm).

The predicted nitrate concentration was 3.0011 ppm, with a maximum deviation of 1.9789 ppm. Based on the 2024 observations, the nitrate concentration ranged from 0.0300 to 8.0000 ppm. The predicted ammonia concentration was 0.0981 ppm, with a maximum deviation of 0.0441 ppm. According to the 2024 observations, the ammonia concentration ranged from 0.0400 to 0.2000 ppm. The predicted oil and grease concentration in river water in East Kalimantan in 2024 was 0.3704 ppm, with a maximum deviation of 0.0465 ppm. The observed oil and grease concentration ranged from 0.3000 to 0.4000 ppm. The predicted water pH was 6.9700, with a maximum deviation of 0.3991. The observed pH ranged from 6.1000 to 7.8500. The predicted TSS (total suspended solids) was 54.1959 ppm, with a maximum deviation of 37.9561 ppm. The observed TSS ranged from 1.2900 to 172.0000 ppm. The predicted water color degree was 83.2593 Pt-Co Units, with a maximum deviation of 49.0609 Pt-Co Units. The observed water color degree ranged from 8.0000 to 185.0000 Pt-Co Units.

Based on the Class 1 river water quality standards presented in Table 1, the covariates total phosphate $\left(X_{2}\right)$, water temperature $\left(X_{3}\right)$, nitrate $\left(X_{5}\right)$, ammonia $\left(X_{6}\right)$, oil and grease $\left(X_{7}\right)$, and water pH $\left(X_{8}\right)$ comply with the quality standards, indicating good water quality. In contrast, the covariates dissolved Fe $\left(X_{1}\right)$, BOD $\left(X_{4}\right)$, TSS $\left(X_{9}\right)$, and water color degree $\left(X_{10}\right)$ do not comply with the quality standards, as their average values exceed the standard limits.

### Estimation of Weibull parameters and distribution testing of the response variable

An important assumption in Weibull regression modeling that must be verified is that the response variable follows a Weibull distribution. Therefore, the initial section of the analysis focuses on testing the distribution pattern of the 2024 DO sample data from rivers in East Kalimantan, which are assumed to follow a Weibull distribution. By performing a right-censored time-to-event data analysis, the distribution pattern of the DO data is tested for values below 6 ppm (the event data), which indicates degradation of RWQ. In this study, an event is defined as RWQ degradation. The maximum likelihood estimators of the Weibull distribution parameters are obtained numerically using the Newton-Raphson iterative algorithm. Accordingly, the CDF of the DO sample data, referring to Eq. (3), can be expressed as follows:

(73) $$\hat{F}\left(y\right)=1-exp\left[-\left(2.6763\;\times 10^{-5}\right)y^{6.81698}\right].$$

Distribution testing in this study was conducted using a nonparametric method, specifically the Kolmogorov-Smirnov test, with the hypotheses formulated as follows:

$H_{0\;}:F\left(y\right)=\hat{F}\left(y\right)$ *(the DO data follow a Weibull distribution with the CDF* $\hat{F}\left(y\right)$ *given in* Eq. (73))

$H_{a\;}:F\left(y\right)\neq \hat{F}\left(y\right)$ *(the DO data do not follow the Weibull distribution)*

*The results of the test statistic and the corresponding critical value are presented in* Table 2*, calculated using GNU Octave.*

**Table 2:** Test statistic and critical value for weibull distribution test.

Test Statistic (D)	D(0.95,21)	Test Result
0.1822	0.2870	H_0_ is accepted

Based on the test statistic presented in Table 2, the statistic D = 0.1822 is less than the critical value $D_{\left(0.95,21\right)}=0.2870$. Therefore, at a 5% significance level, the test concludes that $H_{0}$ cannot be rejected. Thus, the 2024 DO sample data from rivers in East Kalimantan are consistent with a Weibull distribution with scale-shape parameterization as given by the CDF in Eq. (73). Consequently, Weibull regression modeling can be applied to the data.

### Parameter estimation of the Weibull regression model

The parameter estimation process was initiated by examining multicollinearity among the covariates. Detecting multicollinearity is crucial to ensure accurate parameter estimation and reliable analytical results. In this study, the variance inflation factor (VIF) was used to assess multicollinearity. The VIF values for all covariates, computed using GNU Octave software, are presented in Table 3.

**Table 3:** VIF values of covariates.

Covariates	VIF Values
Dissolved Fe (*X*_1_)	1.6682
Total Phosphate (*X*_2_)	1.1200
Water Temperature (*X*_3_)	2.9975
BOD (*X*_4_)	2.8015
Nitrate (*X*_5_)	2.1480
Ammonia (*X*_6_)	1.5686
Oil and Grease (*X*_7_)	2.5108
Water pH (*X*_8_)	3.0182
TSS (*X*_9_)	1.4366
Water Color Degree (*X*_10_)	2.5899

The VIF values for all the covariates, as shown in Table 3, are less than 10, indicating that no multicollinearity exists among the covariates. This result further confirms that the Weibull regression model for the DO data can include all ten covariates listed in Table 3.

The parameter estimation results presented in this study correspond solely to the best WR model. The best WR was selected using a backward elimination approach, in which non-significant covariates (p-value > 0.05) were sequentially removed. The criterion for selecting the best model was a WR model that provides a good fit, achieves the lowest BIC, and includes the largest number of significant covariates (p-value ≤ 0.05).

Based on the selection results, the best WR model includes four covariates: dissolved Fe $\left(X_{1}\right)$, total phosphate $\left(X_{2}\right)$, water temperature $\left(X_{3}\right)$, and BOD $\left(X_{4}\right)$. This optimal WR model yields the minimum BIC value of 32.9160, with three covariates ($X_{1},\;X_{3}$, and $X_{4}$) being significant at the 0.05 level. Although total phosphate $\left(X_{2}\right)$ is not significant at the 0.05 level, it is significant at the 0.10 level. Parameter estimation for the best WR model was performed using the MLE method, and the corresponding log-likelihood function is given in Eq. (17).

Using a right-censored time-to-event modeling approach and referring to the lower bound of the DO quality standard for Class I river water (used as a drinking water source), the response variable (DO) data $y_{i}$ in this study consist of both complete and right-censored observations, with a cutoff value of 6. The event or censoring status is classified into two categories. For the i-th observation, if $y_{i}<6$, the response variable is considered uncensored with $\delta_{i}=1$, indicating that the i-th observation experiences the event, i.e., the RWQ at that point degrades to a poor-quality level. Conversely, if $y_{i}\geq 6$ the response is treated as right-censored with $y_{i}=6\;$and $\delta_{i}=0$, indicating that the observation is censored (survives), meaning that the RWQ at that point remains at a good-quality level.

The maximum likelihood (ML) estimator, denoted by $\hat{\varphi }$*, is the value that maximizes* the log-likelihood function, which incorporates the censoring status, as defined in Eq. (17). This estimator can be obtained by solving the nonlinear equation in Eq. (18). The estimated φ *is computed* numerically using the Newton-Raphson iterative algorithm given in Eq. (22). Parameter estimation was performed using GNU Octave software, and the resulting ML estimator values are presented in Table 4.

**Table 4:** Maximum likelihood (ML) estimates of the WR model parameters.

Parameter	γ	$b_{0}$	$b_{1}$	$b_{2}$	$b_{3}$	$b_{4}$
**ML Estimator**	19.8865	-72.7466	2.0556	30.1975	0.8769	2.5021

Based on the ML estimator presented in Table 4 and considering the invariance property, the WR models for the 2024 DO data of East Kalimantan river water can be derived as follows. The Weibull survival regression model with four covariates, as referred to in Eq. (8), is expressed as:

(74) $$\begin{array}{c} \hat{S}\left(y,x\right)=P\left(Y>y|x\right)=\text{exp}\left[-y^{\hat{\gamma }}\text{exp}\left({\hat{b}}^{T}x\right)\right]=\text{exp}\left[-y^{\hat{\gamma }}\text{exp}\left({\hat{b}}_{0}+{\hat{b}}_{1}X_{1}+{\hat{b}}_{2}X_{2}+{\hat{b}}_{3}X_{3}+{\hat{b}}_{4}X_{4}\right)\right]\\ =\text{exp}\left[-y^{19.8865}\text{exp}\left(-72.7466+2.0556X_{1}+30.1975X_{2}+0.8769X_{3}+2.5021X_{4}\right)\right], \end{array}$$

where y denotes the river water DO concentration, $X_{1}$ represents dissolved Fe, $X_{2}$ represents total phosphate, $X_{3}$ represents water temperature, and $X_{4}$ represents BOD. The corresponding Weibull cumulative distribution regression model, referring to Eq. (9), is expressed as:

(75) $$\begin{array}{c} \hat{F}\left(y,x\right)=P\left(Y\leq y|x\right)=1-\hat{S}\left(y,x\right)=1-\text{exp}\left[-y^{19.8865}\text{exp}\left(-72.7466+2.0556X_{1}+30.1975X_{2}+0.8769X_{3}+2.5021X_{4}\right)\right]. \end{array}$$

The Weibull hazard regression model referring to Eq. (10) is expressed as:

(76) $$\hat{h}\left(y,x\right)=19.8865y^{18.8865}\text{exp}\left(-72.7466+2.0556X_{1}+30.1975X_{2}+0.8769X_{3}+2.5021X_{4}\right),$$

and the Weibull regression model for mean, based on Eq. (11) can be expressed as:

(77) $${\hat{\mu }}_{Y}\left(x\right)=0.9734\exp\left(3.6581-0.1034X_{1}-1.5185X_{2}-0.0441X_{3}-0.1258X_{4}\right).$$

*Based on the event definition of the response variable (DO) used in this study, the WR models presented in* Eqs. (74) *– (77) represent the statistical models of river water quality (RWQ) in East Kalimantan for 2024. Specifically, the Weibull survival regression model in* Eq. (74) *represents the probability of RWQ improvement; the Weibull cumulative distribution regression model in* Eq. (75) *describes the probability of RWQ degradation; the Weibull hazard regression model in* Eq. (76) *characterizes the RWQ degradation rate; and the Weibull mean regression provided in* Eq. (77) *represents the expected DO level of river water in East Kalimantan in 2024*.

### Assessment of Weibull regression parameters

*The statistical models for RWQ expressed in* Eqs. (74) *– (77) were found to be adequate and provided a good fit to the data. This was confirmed through regression parameter testing, conducted both simultaneously and individually, as follows. The hypotheses for the simultaneous testing of the regression parameters, as described in* Eq. (29), *are:*

$H_{0\;}:\;b_{1}=b_{2}=b_{3}=b_{4}=0$ *(the statistical model for RWQ does not fit),*

$H_{a}:$ *At least one* $b_{k}\neq 0,$ *for* k=1,2,3,4 *(the statistical model for RWQ fits).*

*The test statistic W is computed according to* Eq. (30) *or* Eq. (32). *The results obtained using GNU Octave software yield W = 28.1509 and a p-value of* $1.6465\;\times 10^{-13}.$ *The test statistic (W = 28.1509) exceeds the critical value* $\chi_{\left(0.95,\;2\right)}^{2}=9.4877$*, and the p-value (*$1.6465\;\times 10^{-13}$*) is less than the significance level* α=5%*. Thus, the simultaneous test suggests rejecting H_0_ at the 5% significance level. Therefore, it can be concluded that the RWQ statistical model (WR model) fits the data, indicating that the covariates* $X_{1},\;X_{2},\;X_{3}$ *and* $X_{4}$ *simultaneously influence RWQ.*

*The second regression parameters evaluation is the partial test, with the hypotheses given in* Eq. (34)*:* $H_{0}:\;b_{k}=0$ *versus* $H_{a}:\;b_{k}\neq 0$*. The test statistic is computed according to* Eq. (35) *or* Eq. (36)*, and the computation results are presented in* Table 5*. Based on the test statistic values presented in* Table 5, *H_0_ is rejected at the 5% significance level for the covariates dissolved Fe* $\left(X_{1}\right)$*, water temperature* $\left(X_{3}\right)$*, and BOD* $(X_{4}).$ *Therefore, these covariates individually affect RWQ in East Kalimantan. This finding is supported by the absolute values of the test statistic* $Q_{k}\;\left(k=1,\;3,\;4\right)$*, including the intercept, which exceed the critical value of* $Z_{0,975}=1.96$*. Moreover, p-values for these three covariates are less than the significance level* α=5%*.*

**Table 5:** Test statistic values for partial testing of WR model parameters.

Covariates	${\hat{b}}_{k}$	SE	$|Q_{k}|$	*p-value*
Intercept	-72.7466	13.7368	5.2957	$1.1854\;\times 10^{-7}$
$X_{1}$	2.0556	0.9588	2.1440	0.0320
$X_{2}$	30.1975	15.7577	1.9164	0.0553
$X_{3}$	0.8769	0.2300	3.8121	0.0001
$X_{4}$	2.5021	0.5411	4.6239	$3.7658\;\times 10^{-6}$

*Meanwhile, the partial test fails to reject H_0_ at the 5% significance level for the covariate total phosphate* $\left(X_{2}\right)$*, indicating that total phosphate does not significantly affect RWQ in East Kalimantan.*

*The results of the simultaneous and partial parameter tests confirm that the WR model (the RWQ statistical model) is adequate and provides a good fit to the data. The indicators supporting model adequacy and goodness of fit include a BIC value of 32.9160, GCV = 0.4130, McFadden* $\;R^{2}=0.8338$ *, and MAPE = 7.0877%, with the latter representing the predictive accuracy of river water DO. Furthermore, to obtain a better-performing model, an extension of the WR model, referred to as the GWWR model, was proposed.*

### Testing spatial heterogeneity of the response variable

The initial stage of the GWWR modeling involves testing the spatial heterogeneity of the response variable, namely the river water DO data from East Kalimantan in 2024. The hypotheses for the spatial heterogeneity test are formulated as follows:

$H_{0}:{\;\sigma }_{1}^{2}=\sigma_{2}^{2}=\cdots =\sigma_{27}^{2}=\sigma^{2}$ (*the DO data are spatially homogeneous*),

$H_{a}$*: At least one* $\sigma_{i}^{2}\neq \sigma^{2},\;i=1,\;2,\;\ldots ,\;27$ *(the DO data are spatially heterogeneous).*

*The test statistic is defined in* Eq. (68)*, and its computation is performed using GNU Octave software. The results indicate rejection of H_0_, suggesting that DO data exhibit spatial heterogeneity. This conclusion is supported by the test statistic value* $F_{g}=5.0364$, *which* exceeds *the critical value* $F_{\left(0.95,(4,22)\right)}=2.8167$, *and by the p-value of 0.0049, which is below* α=0.05*. Therefore, the null hypothesis H_0_ is rejected. Given that the DO data (the response variable) exhibit spatial heterogeneity, GWWR modeling is appropriate for further analysis.*

### Geographically weighted Weibull regression for dissolved oxygen data

Parameter estimation of the geographically weighted Weibull regression (GWWR) model is conducted at each observation location using the MLE method. This method combines the parameter estimation approach of the GWR model with WR regression for right-censored time-to-event data, resulting in a nonlinear system of log-likelihood equations that incorporates both spatial weights and the censoring status (as defined in Eqs. (48)-(49)). Consequently, the ML estimator must be obtained numerically.

The parameter estimation procedure of the GWWR model at the i-th observation location includes computing the Euclidean distances between observation points, determining the bandwidth, calculating the spatial weights based on Eq. (43), and applying the Newton-Raphson iterative algorithm as described in Eq. (53) to obtain the ML estimator. This process is repeated for different bandwidths until the optimal bandwidth at the i-th observation point is identified, corresponding to the minimum BIC as defined in Eq. (45). The GWWR model estimation at each observation location produces 27 local WR models, referred to as the local RWQ statistical models for East Kalimantan in 2024. The results of parameter estimation and the corresponding test statistics for the GWWR model parameters, obtained using GNU Octave software, are presented in Table 6. Among the 27 estimated GWWR models, Table 6 shows only five, corresponding to the RWQ statistical models at the 1st, 9th, 21st, 22nd, and 24th observation locations. The names of these observation locations are listed in Table 7.

**Table 6:** ML estimator and test statistic values for the RWQ model at five observation points.

*i*	Parameter	$\gamma (u_{i})$	$b_{0}\left(u_{i}\right)$	$b_{1}\left(u_{i}\right)$	$b_{2}\left(u_{i}\right)$	$b_{3}\left(u_{i}\right)$	$b_{4}\left(u_{i}\right)$	$a_{i}^{*}\;\&\;X_{k}^{*}$
1	**Estimator**	33.2416	-117.3854	3.8866	74.6105	1.1213	4.9403	1.2510 $X_{2},X_{3},X_{4}$
	$|W_{k}\left(u_{i}\right)|$	-	3.0428	1.7490	1.9636	2.3731	2.9945	
	***p-value***	-	0.0023	0.0803	0.0496	0.0176	0.0027	
	$R_{S}\left(x_{ik}\right)$	-	-	-	0.5154	0.2903	0.6825	
	$R_{F}\left(x_{ik}\right)$	-	-	-	1.5924	1.8675	1.3880	
	$R_{h}\left(x_{ik}\right)$	-	-	-	2.1088	1.0113	1.0506	
	$R_{\mu }\left(x_{ik}\right)$	-	-	-	0.1060	0.9668	0.8619	
9	**Estimator**	19.8865	-72.7466	2.0556	30.1975	0.8769	2.5021	+∞ $X_{1},X_{3},X_{4}$
	$|W_{k}\left(u_{i}\right)|$	-	5.2957	2.1440	1.9164	3.8121	4.6239	
	***p-value***	-	0.0000	0.0320	0.0553	0.0001	0.0000	
	$R_{S}\left(x_{ik}\right)$	-	-	0.9815	-	0.8914	0.9770	
	$R_{F}\left(x_{ik}\right)$	-	-	1.2170	-	2.2721	1.2697	
	$R_{h}\left(x_{ik}\right)$	-	-	1.0208	-	1.0088	1.0253	
	$R_{\mu }\left(x_{ik}\right)$	-	-	0.9018	-	0.9569	0.8818	
21	**Estimator**	22.3654	-93.0130	1.8941	4.5270	1.4773	2.4872	0.6280 $X_{3},X_{4}$
	$|W_{k}\left(u_{i}\right)|$	-	3.8408	1.6792	0.1520	2.7991	3.1230	
	***p-value***	-	0.0001	0.0931	0.8792	0.0051	0.0018	
	$R_{S}\left(x_{ik}\right)$	-	-	-	-	0.1732	0.8638	
	$R_{F}\left(x_{ik}\right)$	-	-	-	-	2.2167	1.2004	
	$R_{h}\left(x_{ik}\right)$	-	-	-	-	1.0149	1.0252	
	$R_{\mu }\left(x_{ik}\right)$	-	-	-	-	0.9361	0.8948	
22	**Estimator**	22.3348	-86.4423	2.2308	21.1723	1.1970	2.6357	1.2339 $X_{1},X_{3},X_{4}$
	$|W_{k}\left(u_{i}\right)|$	-	4.3608	2.0449	0.9575	3.2657	3.6187	
	***p-value***	-	0.0000	0.0409	0.3383	0.0011	0.0003	
	$R_{S}\left(x_{ik}\right)$	-	-	0.6864	-	0.0309	0.6350	
	$R_{F}\left(x_{ik}\right)$	-	-	1.0894	-	1.2763	1.1041	
	$R_{h}\left(x_{ik}\right)$	-	-	1.0226	-	1.0120	1.0267	
	$R_{\mu }\left(x_{ik}\right)$	-	-	0.9049	-	0.9478	0.8887	
24	**Estimator**	22.2354	-92.8350	1.8047	6.2202	1.4830	2.4598	0.6650 $X_{3},X_{4}$
	$|W_{k}\left(u_{i}\right)|$	-	-3.8101	1.6030	0.2219	2.7584	3.0446	
	***p-value***	-	0.0001	0.1089	0.8244	0.0058	0.0023	
	$R_{S}\left(x_{ik}\right)$	-	-	-	-	0.7421	0.9759	
	$R_{F}\left(x_{ik}\right)$	-	-	-	-	3.8180	1.2636	
	$R_{h}\left(x_{ik}\right)$	-	-	-	-	1.0149	1.0249	
	$R_{\mu }\left(x_{ik}\right)$	-	-	-	-	0.9355	0.8953

**Table 7:** Grouping of RWQ statistical models based on influencing factors.

No	The Influencing Factors	The River Name and the Observation Point (i)	Number of Locations
1	$X_{3},\;X_{4}$	*7) Mahakam River at Nyan point, Mahakam Hulu District; 13) Santan River at Marang Kayu Bridge point, Kutai Kartanegara District; 14) Santan River at Santan Tengah Bridge point, Kutai Kartanegara District; 16) Kedang Kepala River at Kedang Kepala Village point, Kutai Kartanegara District; 17) Kedang Kepala River at Siran Village point, Kutai Kartanegara District; 18) Belayan River at Sebelimbingan Village point, Kutai Kartanegara District; 19) Belayan River at Muara point, Kutai Kartanegara District; 20) Mahakam River at Bloro point, Kutai Kartanegara District; 21) Mahakam River at Kumala Island point, Kutai Kartanegara District; 23) Mahakam River at Palaran point, Samarinda City; 24) Mahakam River at Kalamur point, Samarinda City; 26) Manggar Besar River at the upstream point, Balikpapan City;, 27) Manggar Besar at the downstream point, Balikpapan City.*	13
2	$X_{1},\;X_{3},\;X_{4}$	*4) Segah River at Keraton Gunung Tambur point, Berau District; 8) Boh River at Muara Boh point, Mahakam Hulu District; 9) Mahakam River at Batoq Kelo point, Mahakam Hulu District; 10) Mahakam River at Long Bagun point, Mahakam Hulu District; 11) Bontang River at Samarinda-Bontang Bridge point; Bontang City; 12) Bontang River at Soekarno-Hatta Bridge point, Bontang City; 15) Mahakam River at Tering Point, Kutai Barat District; 22) Mahakam River at front of the Governor’s office of East Kalimantan Province point, Samarinda City; 25) Mahakam River at Anggana point, Kutai Kartanegara District.*	9
3	$X_{2},\;X_{3},\;X_{4}$	*1) Segah River at at Gunung Tabur Bridge point, Berau District; 2) Kelay River at Keraton Sambaliung point, Berau District; 3) Segah River at Hilir Berau Coal point, Berau District; 5) Kelay River at Kantor Bupati Berau point, Berau District; 6) Segah River at the upstream point of PT BBE, Berau District.*	5

*Notes:*$X_{1}:$*dissolved Fe;*$X_{2}:$*total phosphate;*$X_{3}:$*water temperature;*$X_{4}:$*BOD*.

Based on Table 7, the 1st observation point shown in Table 6 represents the Segah River water sampling location at Gunung Tabur Bridge in Berau District. The 9th observation point corresponds to the Mahakam River water sampling site at Batoq Kelo in Mahakam Hulu District. The 21st observation point refers to the Mahakam River water at Kumala Island in Kutai Kartanegara District, while the 22nd observation point represents the sampling site in front of the East Kalimantan Provincial Governor’s Office in Samarinda City. Finally, the 24th observation point corresponds to the Mahakam River sampling site at Kalamur in Samarinda City. Table 6 also presents the absolute values of the $W_{k}\left(u_{i}\right)$ statistic, the p-values, the ratio values $\left(R_{S},\;R_{F},\;R_{h},\;R_{\mu }\right)$, the optimum bandwidth $\left(a_{i}^{*}\right)$, and the influencing covariates $\left(X_{k}^{*}\right)$. These statistics serve as references for partial parameter testing and for interpreting the RWQ statistical model.

The local RWQ statistical model consists of four spatial Weibull regression models. These include: a spatial Weibull survival regression model, which represents *the probability of RWQ improvement* at an observation location, as expressed in Eq. (38); a spatial Weibull cumulative distribution regression model, which represents the probability of RWQ degradation at an observation location, as expressed in Eq. (39); a spatial Weibull hazard regression model, which represents the RWQ degradation rate at an observation location, as expressed in Eq. (40); and a spatial Weibull mean regression model, which represents the mean DO level of river water at an observation location, as expressed in Eq. (41).

For example, the local model parameters of the RWQ at the 22nd observation point (Mahakam River, in front of the Governor’s Office of East Kalimantan Province, Samarinda City) are presented in Table 6 (i = 22). Referring to Eqs. −(41), the Mahakam River water quality models at this location are given as follows. In particular, the model representing the probability of water quality improvement at this observation (Eq. (38)) is:

(78) $$\hat{S}\left(y_{22},x_{22}\right)=P\left(Y>y_{22}|x_{22}\right)=\text{exp}\left[-y_{22}^{22.3348}\text{exp}\left(-86.4423+2.2308x_{22,1}+21.1723x_{22,2}+1.1970x_{22,3}+2.6357x_{22,4}\right)\right].$$

The model representing the probability of Mahakam River water quality degradation at the observation point in front of the East Kalimantan Provincial Governor’s Office (Eq. (39)) is:

(79) $$\hat{F}\left(y_{22},x_{22}\right)=P\left(Y\leq y_{22}|x_{22}\right)=1-\hat{S}\left(y_{22},x_{22}\right)=1-\text{exp}\left[-y_{22}^{22.3348}\text{exp}\left(-86.4423+2.2308x_{22,1}+21.1723x_{22,2}+1.1970x_{22,3}+2.6357x_{22,4}\right)\right].$$

The model representing the Mahakam River water quality degradation rate at the observation point in front of the East Kalimantan Provincial Governor’s Office (Eq. (40)) is:

(80) $$\hat{h}\left(y_{22},x_{22}\right)=22.3348y^{21.3348}\text{exp}\left(-86.4423+2.2308x_{22,1}+21.1723x_{22,2}+1.1970x_{22,3}+2.6357x_{22,4}\right),$$

*and the model representing the mean DO level of Mahakam River water at the observation point in front of the East Kalimantan Provincial Governor’s Office (*Eq. (41)*) is:*

(81) $${\hat{\mu }}_{Y}\left(x_{22}\right)=0.9761\exp\left(3.8703-0.0999x_{22,1}-0.9480x_{22,2}-0.0536x_{22,3}-0.1180x_{22,4}\right).$$

The GWWR modeling of river water DO in East Kalimantan in 2024, with ML estimators at several observation points presented in Table 6, was found to be adequate and provided a good fit. Model adequacy and goodness-of-fit were confirmed through parameter testing, which included similarity testing between the GWWR (local) model and the WR (global) model, as well as simultaneous and partial parameter tests.

### Test of similarity between the GWWR model and the Weibull regression model

The hypotheses for the similarity test between the GWWR (local) model and the WR (global) model are formulated as follows:

$H_{0}:\;b_{k}\left(u_{i}\right)=b_{k},$ for k=1,2,3,4;i=1,2,…,27 (the fitted model is the WR (global) model)

$H_{a}:At\;least\;one\;b_{k}\left(u_{i}\right)\neq b_{k},$ for k=1,2,3,4;i=1,2,…,27 (the fitted model is the GWWR model).

*The test statistic is given in* Eq. (59), *and the computation results obtained using GNU Octave software are* G=258.9119 *with a p-value of* $2.0650\;\times 10^{-14}$*. Based on these results, the hypothesis test suggests rejecting H_0_ at the 5% significance level for the similarity test between the GWWR and the WR models. This conclusion is supported by the fact that the test statistic G exceeds the critical value* $\chi_{\left(0.95,108\right)}^{2}=133.2569$*, and the p-value is less than the significance level* α=0.05*. Therefore, the similarity test indicates that the GWWR model and the WR model are not identical, with the GWWR model (local model) providing a better fit.*

### Simultaneous test of geographically weighted Weibull regression model parameters

The hypotheses for the simultaneous parameter test are formulated as follows:

$H_{0}:\;b_{1}\left(u_{i}\right)=b_{2}\left(u_{i}\right)=b_{3}\left(u_{i}\right)=b_{4}\left(u_{i}\right)=0,$ for i=1,2,…,27 (the GWWR model is not appropriate)

$H_{a}:At\;least\;one\;b_{k}\left(u_{i}\right)\neq 0,$ for k=1,2,3,4;i=1,2,…,27 (the GWWR model is appropriate).

The test statistic is given in Eq. (64), and the results obtained using GNU Octave software are $G_{w}=\;910610.5112$ and a p-value of 0.0000. The test statistic $G_{w}=\;910610.5112\;$exceeds the critical value $\chi_{\left(0.95,108\right)}^{2}=126.5741,$ and the p-value of 0.0000 is less than the significance level α=5%. Therefore, the test results indicate that the parameters should be tested simultaneously and that H_0_ should be rejected, leading to the conclusion that the local RWQ statistical model, namely the GWWR model, is appropriate.

### Partial test of the geographically weighted Weibull regression model

The final adequacy assessment of the GWWR model is the partial parameter test. The hypotheses for the partial parameter test for $b_{k}\left(u_{i}\right)\;$where k=1,2,3,4 and i=1,2,…,27, are formulated as follows:

$H_{0}:\;b_{k}\left(u_{i}\right)=0,\;$(the covariate $X_{k}$ does not affect the RWQ statistical model)

$H_{a}:\;b_{k}\left(u_{i}\right)\neq 0$, (the covariate $X_{k}$ affects the RWQ statistical model)

The test statistic is given in Eq. (65). The computed absolute values of the Wald statistic $|W_{k}\left(u_{i}\right)|$, and the corresponding p-values are presented in Table 6. Based on the partial parameter test, the local factors influencing RWQ at each observation location can be identified.

For example, the parameters of the Mahakam River water-quality statistical model at the 22nd observation point (located in front of the Governor’s Office of East Kalimantan Province) are presented in Table 6 (i = 22). The RWQ statistical models are expressed by Eqs. (78) – (81), and the influencing factors include dissolved Fe ($X_{2}$), water temperature ($X_{3}$), and BOD ($X_{4}$). This is supported by the values of $|W_{k}\left(u_{22}\right)|\;$for the parameters $b_{1}\left(u_{22}\right),\;b_{3}\left(u_{22}\right)$, and $b_{4}\left(u_{22}\right)$, which exceed the critical value $Z_{0.975}=1.96$, as well as their corresponding p-values, which are lower than the significance level α=0.05. Therefore, the parameter tests reject $H_{0}$ for $b_{1}\left(u_{22}\right),\;\;b_{3}\left(u_{22}\right)$, and $b_{4}\left(u_{22}\right)$, indicating that the covariates $X_{1}$, $X_{3}$ and $X_{4}$ individually influence the Mahakam River water quality at the observation point in front of the Governor’s Office of East Kalimantan Province in Samarinda City.

Meanwhile, the covariate total phosphate ($X_{2}$) does not influence the Mahakam River water quality at the observation point in front of the Governor’s Office of East Kalimantan Province. This conclusion is based on the absolute value of the Wald statistic for parameter $b_{2}\left(u_{22}\right)$, which is below the critical value, and on its corresponding p-value, which exceeds the 5% significance level, as shown in Table 6. Similarly, partial testing of the GWWR model parameters at other observation points can be conducted in the same manner.

**Fig. 2:**
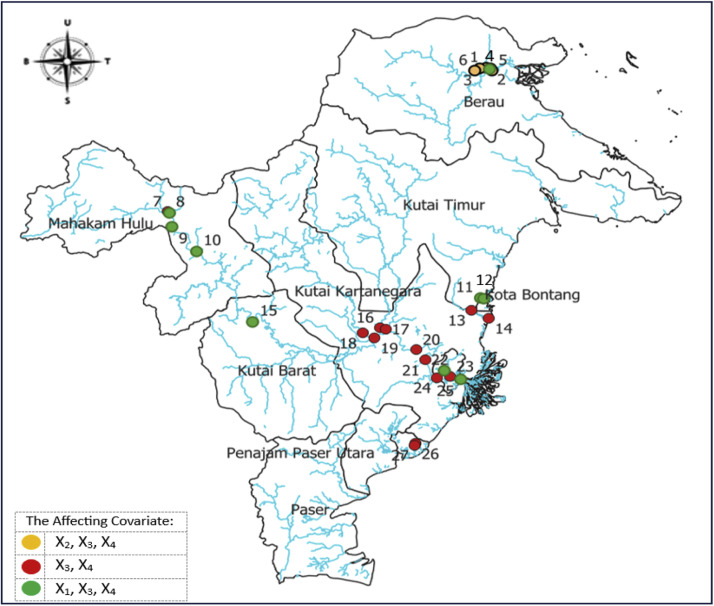
Spatial distribution of factors influencing river water quality (RWQ) in East Kalimantan, 2024.

By partially testing the GWWR model parameters at each observation point, the local factors influencing the river water quality (RWQ) can be identified, and a local factor map of RWQ in East Kalimantan can be generated. This local factor mapping provides valuable information for the people of East Kalimantan to help maintain good river water quality and to actively participate in preventing river pollution. It can also be recommended to the government as a basis for formulating river pollution prevention policies. Based on the identified influencing factors, the RWQ statistical model in East Kalimantan can be classified into three groups, as presented in Table 7 and Fig. 2.

By analyzing the RWQ influencing-factor mapping presented in Table 7, both global and local influencing factors can be identified. The global influencing factors are water temperature $(X_{3}$) and BOD $\left(X_{4}\right)$, whereas the local influencing factors are dissolved Fe $\left(X_{1}\right)$ and total phosphate $\left(X_{2}\right)$. The spatial distribution of these influencing factors and the grouping of RWQ statistical models based on them are illustrated in Fig. 2. This Fig. visualizes the RWQ influencing factors across East Kalimantan in 2024, generated using QGIS software. As shown in Fig. 2, rivers in East Kalimantan are represented by blue lines, while red, green, and orange dots indicate the observation points. The numbers assigned to each dot correspond to the observation site names listed in Table 7.

Referring to Table 7, the RWQ statistical models represented by the red points in Fig. 2 are classified as the first group of RWQ models, with water temperature $\left(X_{3}\right)$ and BOD $\left(X_{4}\right)$ identified as their influencing factors. Based on Fig. 2, these first-group RWQ models are generally located in Kutai Kartanegara District, Balikpapan City, and several observation sites in Samarinda City. This group comprises 13 models, corresponding to locations numbered 7, 13, 14, 16, 17, 18, 19, 20, 21, 23, 24, 26, and 27, as listed in Table 7.

The RWQ statistical models at the green observation points, as shown in Fig. 2, are classified as the second group of RWQ models, with dissolved Fe $\left(X_{1}\right)$, water temperature $(X_{3}),$ and BOD $\left(X_{4}\right)$ identified as the influencing factors. Based on Fig. 2, the second-group RWQ models are generally located along the Mahakam River, particularly at observation sites in Mahakam Hulu District, Kutai Barat District, and Samarinda City. This group comprises nine RWQ models, corresponding to locations numbered 4, 8, 9, 10, 11, 12, 15, 22, and 25, as listed in Table 7.

Referring to Table 6, Table 7, the RWQ statistical models represented by the orange observation points in Fig. 2 are classified as the third group of RWQ models, with total phosphate $\left(X_{2}\right)$, water temperature $\left(X_{3}\right)$, and BOD $\left(X_{4}\right)$ identified as the influencing factors. This group includes five RWQ models located at observation sites in the northern part of East Kalimantan (Berau District), corresponding to locations numbered 1, 2, 3, 5, and 6, as listed in Table 7.

Parameter testing, comprising similarity model testing, simultaneous parameter testing, and partial parameter testing, confirmed that the GWWR modeling of DO data from East Kalimantan river water in 2024 was adequate and produced a well-fitted model. The best model between GWWR model (local RWQ statistical model) and WR model (global RWQ model) was selected based on the goodness-of-fit measures, including BIC, GCV, McFadden $R^{2}$, and MAPE, as shown in Table 8.

**Table 8:** Measures of goodness-of-Fit for RWQ models.

Model	The Adequacy Model Measurer			
	BIC	GCV	McFadden-R^2^	MAPE (%)
WR (global RWQ)	32.9160	0.4130	0.8338	7.0877
GWWR (local RWQ)	11.0808	0.2725	0.9071	5.2349

The goodness-of-fit measures presented in Table 8 indicate that the GWWR model (local RWQ model) outperforms the WR model (global RWQ model). This conclusion is supported by performance metrics such as BIC, GCV, and MAPE, all of which are smaller for the GWWR model than for the WR model. Additionally, the McFadden R^2^ value of the GWWR model is higher than that of the WR model. The MAPE values shown in Table 8 represent the accuracy of DO predictions obtained using the spatial Weibull regression model for the mean.

### Interpretation of the geographically weighted Weibull regression model

**Fig. 3:**
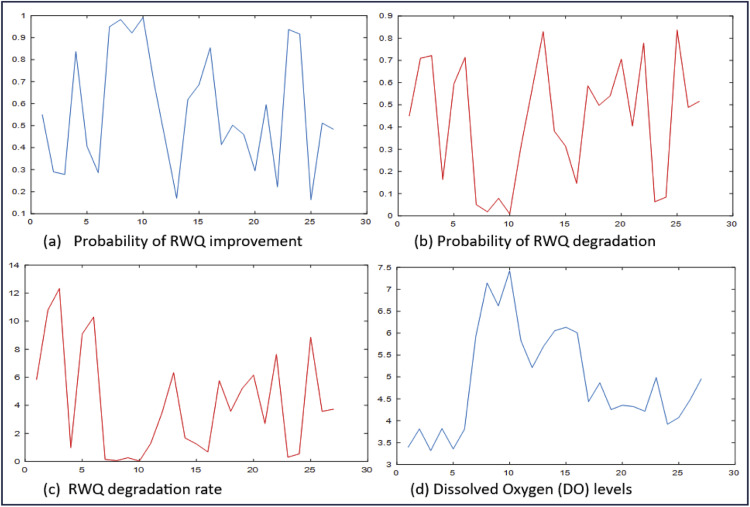
Statistical measures of RWQ across the observation points in East Kalimantan, 2024.

As mentioned in the previous section, the GWWR model is a local RWQ statistical model for East Kalimantan, defined at each observation location. The RWQ statistical model, expressed in Eqs. (78)–(81), consists of four local components: the probability model for river water quality improvement $S(y_{i},x_{i})$, the probability model for river water quality degradation $F(y_{i},x_{i})$, the degradation rate model $h(y_{i},x_{i})$, and the mean DO model $\mu (x_{i})$. By applying this model to the sample data, the quantities $\hat{S}\left(y_{i},x_{i}\right)$, $\hat{F}\left(y_{i},x_{i}\right)$, $\hat{h}\left(y_{i},x_{i}\right)$, and $\hat{\mu }\left(x_{i}\right)$ can be obtained at each observation point. These quantities represent statistical measures of RWQ and provide valuable information on RWQ conditions in East Kalimantan. The RWQ characteristics were evaluated using statistical measures derived from the GWWR model. RWQ measures across all observation points are presented in Fig. 3, while the measures at selected observation points are presented in Table 9.

**Table 9:** Statistical Measures of RWQ for Five Observation Points.

Observation Point (i)	Statistical Measures of River Water Quality			
	$\hat{S}\left(y_{i},x_{i}\right)$	$\hat{F}\left(y_{i},x_{i}\right)$	$\hat{h}\left(y_{i},x_{i}\right)$	$\hat{\mu }\left(x_{i}\right)$ (ppm)
1	0.5500	0.4500	5.8445	3.3961
9	0.9213	0.0787	0.2716	6.6231
21	0.5954	0.4046	2.6970	4.3223
22	0.2219	0.7781	7.6428	4.2167
24	0.9162	0.0838	0.5408	3.9202
Regional Average Values	0.5718	0.4282	4.1723	4.9047

Table 9 presents the statistical measures of RWQ at five observation points, namely the 1st, 9th, 21st, 22nd, and 24th points. The table also includes the regional average of these measures, shown in the last row. Based on the values in the last row, RWQ in East Kalimantan in 2024 is characterized by a probability of quality improvement of 0.5718, a probability of degradation of 0.4282, a degradation rate of 4.1723 locations per 1-ppm change in DO concentration (approximately 4 locations per 1 ppm), and an average DO level of 4.9047 ppm. According to the RWQ map generated using the GWWR model, the overall RWQ in East Kalimantan in 2024 tends to degrade; however, the quality varies across observation points. The spatial variation of RWQ based on these statistical measures is illustrated in Fig. 3. Similarly, the characteristics of RWQ based on the statistical measures at selected observation points are described as follows:

For example, the statistical measures presented in the second row of Table 9 (i = 9) show the RWQ at the observation point in Batoq Kelo, Mahakam Hulu District. The RWQ at this observation point is characterized by a probability of quality improvement of 0.9213 (very high), a probability of degradation of 0.0787 (very low), a degradation rate of 0.2716 locations per 1 ppm change in DO concentration (approximately 3 locations per 10 ppm; low), and an average DO level of 6.6231 ppm (high). Based on these statistical measures, the RWQ at this observation point in Batoq Kelo can be classified as good.

Referring to Table 7 and Fig. 2, the 22nd observation point is located in the Mahakam River in front of the Governor’s Office of East Kalimantan Province in Samarinda City. The statistical measures of RWQ presented in the fourth row of Table 9 confirm that the probability of RWQ improvement is 0.2219 (low), corresponding to a probability of RWQ degradation of 0.7781 (high). The RWQ degradation rate is relatively fast, at 7.6428 locations per 1 ppm DO (approximately 8 locations per 1 ppm DO), while the predicted DO level at the 22nd observation point is 4.2167 ppm (less than 6 ppm). Based on these four statistical measures, the RWQ at this observation point can be considered degraded. Similarly, the characteristics of RWQ at other observation points can be described using the same approach.

The interpretation of the GWWR model can also be performed based on the ratio values of the RWQ statistical model before and after increasing the covariate values. For example, the interpretation of the RWQ model at the 9th observation point, corresponding to the Mahakam River at Batoq Kelo, Mahakam Hulu District, is as follows. Referring to Table 6 (i=9), the covariates affecting the RWQ model at this observation point were dissolved Fe $\left(X_{1}\right)$, water temperature $\left(X_{3}\right)$, and BOD $\left(X_{4}\right)$. The optimal bandwidth of the local RWQ model at the 9th observation point was found to be +∞ (very large positive number), indicating that this model at this observation point is identical to the global model, as expressed in Eqs. (74)-(77) with ML estimator displayed in Table 4, or in Table 6 (i=9). Based on the Weibull survival regression model in Eq. (74), the ratio value $R_{S}\left(x_{9,1}\right)=0.9815$ in Table 6 (i=9) indicates that an increase of 1 ppm in dissolved Fe decreases the probability of RWQ improvement at this observation point to 0.9815 times its original value, corresponding to a decrease of 1.85%. Similarly, the ratio value $R_{F}\left(x_{9,1}\right)=1.2170$ shows that an increase of 1 ppm in dissolved Fe increases the probability of RWQ degradation at the same observation point to 1.2170 times its original value, corresponding to an increase of 21.70%.

**Fig. 4:**
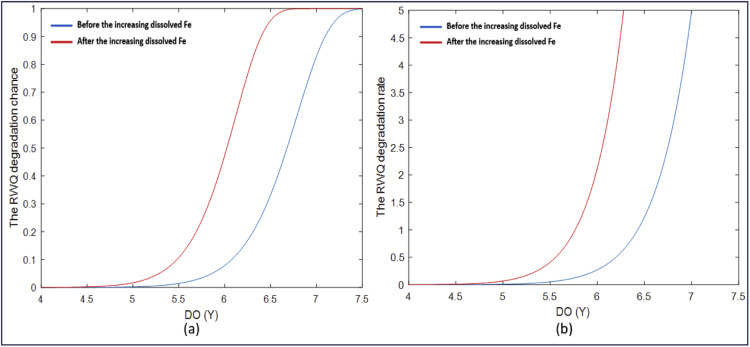
(a) RWQ degradation probability before and after a 1 ppm increase in dissolved iron $\left(Fe,\;X_{1}\right)$ (b) RWQ degradation rate before and after a 1 ppm increase in dissolved iron $\left(Fe,\;X_{1}\right)$.

The ratio value $R_{h}\left(x_{9,1}\right)=1.0208,\;$shown in Table 6 (i=9), indicates that an increase of 1 ppm in dissolved Fe accelerates water quality degradation at the Batoq Kelo observation point in the Mahakam River by a factor of 1.0208 (i.e., an increase of 2.08%). Similarly, $R_{\mu }\left(x_{9,1}\right)=0.9018\;$indicates that an increase of 1 ppm in dissolved Fe reduces the DO level at this point to 0.9018 times its original value, corresponding to a decrease of 9.82%. Graphically, the increase in the water quality degradation probability, $\hat{F}\left(y,x_{9}\right)$, and the increase in the degradation rate, $\hat{h}\left(y,x_{9}\right)$, after adding 1 ppm of dissolved Fe are illustrated in Figs. 4(a) and 4(b), respectively.

*The red curve in* Fig. 4*(a) represents the graph of* $\hat{F}\left(y,x_{9}|x_{9,1}+1\right)$*, illustrating the water quality degradation probability at the Batoq Kelo observation point in the Mahakam River after a 1 ppm increase in dissolved Fe. In contrast, the blue curve represents* $\hat{F}\left(y,x_{9}\right)$*, showing the degradation probability before the increase in dissolved Fe. Similarly, the red curve in* Fig. 4*(b) represents* $\hat{h}\left(y,x_{9}|x_{9,1}+1\right)$*, illustrating the water quality degradation rate at the Batoq Kelo observation point in the Mahakam River after a 1 ppm increase in dissolved Fe, while the blue curve shows the degradation rate before the increase. In* Figs. 4*(a) and 4(b), the red curves lie above the blue curves, indicating that an increase in dissolved Fe elevates the degradation probability and accelerates the degradation rate at the Batoq Kelo observation point. Referring to the second row of* Table 9*, together with the ratio values* $R_{F}\left(x_{9,1}\right)$ *and* $R_{h}\left(x_{9,1}\right)$ *in* Table 6 *(*i=9*), and* Figs. 4*(a) and 4(b), it can be concluded that the observed increase in degradation probability is consistent with the acceleration of the degradation rate. This indicates that higher degradation probabilities correspond to faster degradation rates.*

*In the same way, the ratio value* $R_{S}\left(x_{9,3}\right)=0.8914$ *in* Table 6 *(*i=9*) indicates that a 1°C increase in water temperature decreases the probability of water quality improvement in the Mahakam River at the Batoq Kelo observation point to 0.8914 times its original value, corresponding to a 10.86% decrease. In contrast, the ratio* $R_{F}\left(x_{9,3}\right)=2.2721$ *shows that a 1°C increase in water temperature raises the Mahakam River water quality degradation probability at the Batoq Kelo observation point by a factor of 2.2721, representing an increase of 127.21%. Similarly, the ratio value* $R_{h}\left(x_{9,3}\right)=1.0088$ *shows that a 1°C increase in water temperature slightly accelerates the water quality degradation rate at the Batoq Kelo observation point of the Mahakam River by a factor of 1.0088, corresponding to an increase of 0.88%. Finally,* $R_{\mu }\left(x_{9,3}\right)=0.9569$ *indicates that the same temperature increase reduces the DO level in the river water at Batoq Kelo to 0.9569 times its initial value, corresponding to a 4.31% decrease.*

**Fig. 5:**
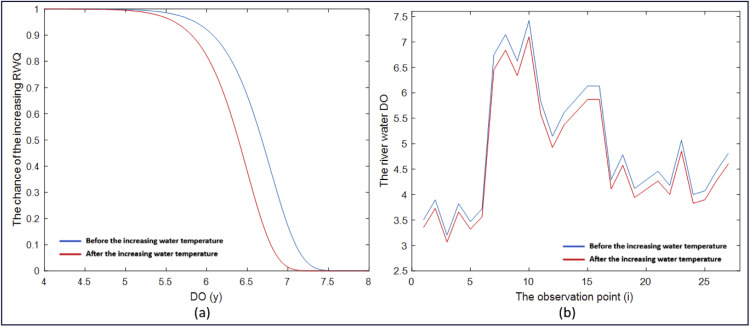
(a) Probability of RWQ improvement before and after a 1°C increase in water temperature $\left(X_{3}\right)$ (b) River water DO levels before and after a 1°C increase in water temperature $\left(X_{3}\right)$.

*Graphically, the decrease in the probability of water quality improvement in the Mahakam River at the Batoq Kelo observation point in Mahakam Hulu District* $\hat{(S}\left(y,x_{9}\right))$ *and the decrease in the river water DO level (*$\hat{\mu }\left(y,x_{9}\right)$*) following a 1°C increase in water temperature are shown in* Figs. 5*(a) and 5(b), respectively.*

The red curve in Fig. 5(a) represents the probability of Mahakam River water quality improvement at the Batoq Kelo observation point after a 1°C increase in water temperature, while the blue curve shows the probability before the increase. Similarly, the red curve in Fig. 5(b) represents the DO level of Mahakam River at the Batoq Kelo observation point after a 1°C increase in water temperature, whereas the blue curve shows the DO level before the increase.

The red curves in Fig. 5(a) and 5(b) lie below the blue curves, confirming that an increase in water temperature decreases the probability of RWQ improvement and reduces the river’s DO level. Referring to Table 6 (i=9), Table 9, and Fig. 5(a) and 5(b), the decrease in RWQ improvement probability is consistent with the decrease in the river’s DO level, indicating that a reduction in RWQ improvement probability corresponds to a decrease in the river’s DO level.

Referring to Table 6 (i=9), the ratio value $R_{S}\left(x_{9,4}\right)=0.9770$ indicates that a 1 ppm increase in BOD reduces the RWQ improvement probability at the Batoq Kelo observation point to 0.9770 times its original value, corresponding to a decrease of 2.30%. The ratio $R_{F}\left(x_{9,4}\right)=1.2697$ shows that the same increase in BOD raises the RWQ degradation probability at the same observation point to 1.2697 times its initial value, representing an increase of 26.97%.

Similarly, the ratio value $R_{h}\left(x_{9,4}\right)=1.0253$ in Table 6 (i=9) indicates that a 1 ppm increase in BOD accelerates the RWQ degradation rate at the Batoq Kelo observation point by 2.53%. Meanwhile, $R_{\mu }\left(x_{9,4}\right)=0.8818$ suggests that the same increase in BOD reduces the river’s DO level at the Batoq Kelo observation point to 0.8818 times its initial value, corresponding to a decrease of 11.82%.

**Fig. 6:**
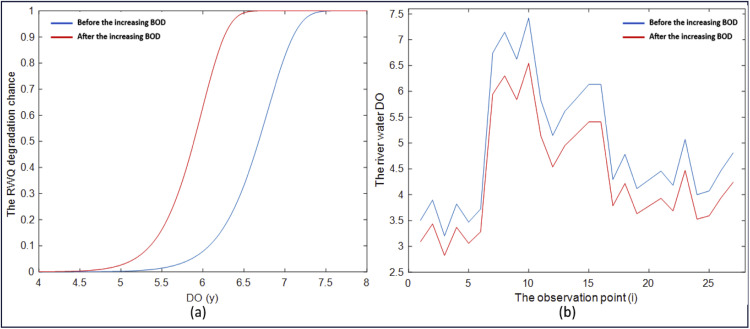
(a) RWQ degradation probability before and after a 1 ppm increase in BOD concentration $\left(X_{4}\right)$ water DO level before and after a 1 ppm increase in BOD concentration $\left(X_{4}\right)$.

Graphically, the increase in RWQ degradation probability and the decrease in DO concentration at the Batoq Kelo observation point (Mahakam Hulu District) following a 1 ppm increase in BOD are illustrated in Fig. 6(a) and 6(b), respectively. As shown in Fig. 6(a), the red and blue curves represent the RWQ degradation probability after and before the 1 ppm BOD increase, respectively. The red curve lies above the blue curve, confirming that an increase in BOD leads to a higher probability of RWQ degradation at the Batoq Kelo observation point.

The red and blue curves in Fig. 6(b) represent the river’s DO level at the Batoq Kelo observation point after and before a 1 ppm increase in BOD, respectively. The red curve lies below the blue curve, confirming that an increase in BOD reduces the river’s DO level at this observation point. Referring to Table 6 (i=9), Table 9, and Fig. 6(a) and 6(b), the increase in RWQ degradation probability corresponds to the decrease in the river’s DO level. In other words, the probability of RWQ degradation increases if and only if the river’s DO decreases.

Referring to the GWWR model interpretation, it can generally be concluded that the RWQ information, comprising statistical measures of the decrease in RWQ improvement probability, the increase in RWQ degradation probability, the acceleration of the degradation rate, and the decrease in DO, is effectively equivalent. That is, each RWQ indicator conveys equivalent information about water quality changes. Based on the RWQ statistical model graphs (local Weibull regression model) shown in Figs. 4−6, it can be observed that the blue curve (before the increase in the covariate value) and the red curve (after the increase) are nearly parallel. This confirms that the RWQ statistical model (GWWR model) follows a proportional hazards structure.

Based on mapping the factors influencing RWQ, several recommendations are provided for the government, industry, and the public regarding RWQ maintenance and pollution prevention. The presence of dissolved Fe is suspected to originate from mud in the water flow or from sand on the riverbed. The mud likely originates from soil erosion, and is carried into the river by rainwater during rainfall events. Managing deforested areas and former mining sites can reduce erosion, thereby preventing increases in dissolved Fe in river water. Reforestation of these areas can further reduce soil erosion and mitigate global warming, which helps maintain the river’s water temperature. The increase in phosphate and BOD in the river is suspected to result from the accumulation of decomposing organic matter. Preventing soil erosion, implementing proper household and industrial waste management, and maintaining a clean watershed can help prevent increases in phosphate and BOD levels.

The conclusions regarding the analysis of the geographically weighted Weibull regression model for river water dissolved oxygen (DO) in East Kalimantan in 2024 are as follows. The GWWR model provides a robust local statistical framework for evaluating river water quality, incorporating four spatially explicit formulations: the spatial Weibull survival regression model, the spatial Weibull cumulative distribution regression model, the spatial Weibull hazard regression model, and the spatial Weibull mean regression model. The model effectively captures and maps spatial variations in river water quality across all observation sites. In East Kalimantan in 2024, river water quality exhibits a general tendency toward degradation. This condition is reflected in several statistical indicators, including a probability of improvement of 0.5718, a probability of degradation of 0.4282, a degradation rate of 4.1723 locations per 1-ppm decrease in DO (approximately 4 locations per 1 ppm), and an average DO concentration of 4.9047 ppm. In addition, the GWWR model successfully identifies the key factors influencing river water quality in East Kalimantan in 2024. These factors operate at both global and local spatial scales. Water temperature and biochemical oxygen demand (BOD) act as global determinants, whereas dissolved iron (Fe) and total phosphate emerge as local factors affecting river water quality.

## Limitations

‘Not applicable’

## Ethics statements

The research data used in this study are secondary data obtained from the 2024 Surface Water Quality Monitoring Analysis Report published by the Environmental Agency of East Kalimantan Province. The dataset consists of 27 sampling points. The variables include the response variable, namely the dissolved oxygen (DO) levels of river water in East Kalimantan (Y), and covariates, namely dissolved iron (Fe) $\left(X_{1}\right)$, total phosphate $\left(X_{2}\right)$, water temperature $\left(X_{3}\right)$, biochemical oxygen demand (BOD) $\left(X_{4}\right)$, nitrate $\left(X_{5}\right)$, ammonia $\left(X_{6}\right)$, oil and grease $\left(X_{7}\right)$, pH or potential of hydrogen $\left(X_{8}\right)$, total suspended solids (TSS) $\left(X_{9}\right)$, and water color degree $\left(X_{10}\right)$.

## CRediT authorship contribution statement

**Suyitno Suyitno**: Conceptualization, Methodology, Supervision, Validation, Writing – Original Draft. **Darnah**: Conceptualization, Formal Analysis, Validation, Supervision. **Memi Nor Hayati, Andrea Tri Rian Dani:** Visualization, Data Analysis, Writing-Review & Editing. **Ika Purnamasari**: Project Administration. **Pratama Yuly Nugraha**: Data Curation, Visualization, Software. **Meiliyani Siringoringo**: Investigation, Writing-Original Draft. **Rito Goejantoro**: Formal Analysis, Validation. **Meirinda Fauziyah**: Investigation, Visualization. **Zabrina Nathania Fauziyah**: Data Curation. **Mislan**: Investigation.

## Declaration of competing interests

The authors declare that they have no known competing financial interests or personal relationships that could have appeared to influence the work reported in this paper.

## Data Availability

Data will be made available on request.
